# Horizontally arranged zinc platelet electrodeposits modulated by fluorinated covalent organic framework film for high-rate and durable aqueous zinc ion batteries

**DOI:** 10.1038/s41467-021-26947-9

**Published:** 2021-11-16

**Authors:** Zedong Zhao, Rong Wang, Chengxin Peng, Wuji Chen, Tianqi Wu, Bo Hu, Weijun Weng, Ying Yao, Jiaxi Zeng, Zhihong Chen, Peiying Liu, Yicheng Liu, Guisheng Li, Jia Guo, Hongbin Lu, Zaiping Guo

**Affiliations:** 1 State Key Laboratory of Molecular Engineering of Polymers, Department of Macromolecular Science, Fudan University, 2005 Songhu Road, 200438 Shanghai, China; 2 School of Materials Science & Engineering, University of Shanghai for Science and Technology, Shanghai, 200093 China; 3 Key Laboratory of Advanced Energy Materials Chemistry (Ministry of Education), College of Chemistry, Nankai University, 300071 Tianjin, China; 4 Yiwu Research Institute of Fudan University, Chengbei Road, 322000 Yiwu, Zhejiang China; 5 Chemical Engineering & Advanced Materials, The University of Adelaide, Adelaide, SA 5005 Australia

**Keywords:** Batteries, Materials chemistry

## Abstract

Rechargeable aqueous zinc-ion batteries (RZIBs) provide a promising complementarity to the existing lithium-ion batteries due to their low cost, non-toxicity and intrinsic safety. However, Zn anodes suffer from zinc dendrite growth and electrolyte corrosion, resulting in poor reversibility. Here, we develop an ultrathin, fluorinated two-dimensional porous covalent organic framework (FCOF) film as a protective layer on the Zn surface. The strong interaction between fluorine (F) in FCOF and Zn reduces the surface energy of the Zn (002) crystal plane, enabling the preferred growth of (002) planes during the electrodeposition process. As a result, Zn deposits show horizontally arranged platelet morphology with (002) orientations preferred. Furthermore, F-containing nanochannels facilitate ion transport and prevent electrolyte penetration for improving corrosion resistance. The FCOF@Zn symmetric cells achieve stability for over 750 h at an ultrahigh current density of 40 mA cm^−2^. The high-areal-capacity full cells demonstrate hundreds of cycles under high Zn utilization conditions.

## Abstract (web summary)

Rechargeable aqueous zinc-ion batteries are promising but the zinc anode suffers from dendrite growth and electrolyte corrosion. Here, the authors develop a fluorinated covalent organic framework film as a protective layer for aqueous zinc anode battery.

## Introduction

Zinc (Zn) anode-based aqueous batteries are attracting tremendous interest owing to Zn’s high theoretical capacity (820 mAh g^−1^), low potential (−0.762 V versus the standard hydrogen electrodes), high natural abundance, low cost, and intrinsic non-flammable advantage over organic-based lithium batteries^1–3^. Unfortunately, previous Zn anodes showed poor reversibility in aqueous electrolytes^1,4,5^. Issues including Zn dendrite formation, continuous parasitic hydrogen evolution reaction (HER), and irreversible by-products, resulting in low Coulombic efficiency (CE), and shortened battery life^6–9^. To stabilize the Zn anode, various strategies including electrolyte optimization (additives^10^ and water-in-salt^1^/gel electrolytes^11^), surface coating materials (e.g., metal-organic frameworks^12^, polyamide^8^, and ZnO networks^6^) and Zn bulk structure engineering (e.g., CNT frameworks^13^ or zinc–aluminum alloy^14^) have been proposed to achieve higher performance Zn anodes. However, there are still some unsolved issues with these strategies, which restricts them to subdued performance levels in Zn batteries. For instance, manipulating previous electrolyte compositions leads to an increase in overall costs^15^, sacrifices rate performance of the batteries owing to their slow ionic conductivity and the HER is only lowered, not eliminated^2^. Interfacial modification layers are effective for suppressing HER^8,16^, however, the huge volume change during repeated Zn plating/stripping can damage protective layers, even peeling them completely off the Zn matrix^17^. Employing conductive 3D hosts could help to realize high-rate Zn deposition^13,18^, but adds additional porosity and weight, thereby reducing the volumetric/gravimetric energy density of the batteries. Therefore, developing alternative techniques to achieve dendrite-free Zn anodes while maintaining fast Zn deposition is urgently needed.

The crystallinity and morphology of Zn electrodeposits dominates the reversibility of Zn plating/stripping^5,19,20^, yet the linkage has often not been considered. Modulating irregularly-shaped Zn to planar Zn electrodeposits is desirable for high reversibility of Zn anodes^5^. The electrodeposition processes of Zn, which involve crystallization, exhibit a direct correlation to the morphology of deposits^20^. Upon plating, the influences of external factors often promote the preferred orientation of Zn grains along a specific crystal plane, thus leading to a specific morphological “texture“^21–23^. The morphology and texture of Zn deposits have been proved to be closely related to additives^22,24–26^, initial substrate composition and texture^5,27,28^, and applied external fields^19,29^. Organic molecules and additives can adsorb on a Zn surface, guiding the Zn deposits to show specific preferred orientation of crystal planes^25,26,30^. For example, the polyethylene-glycolin electrolytes^25^ make the Zn deposits show a preferred orientation exposure of (002) and (103) planes, which mitigates dendrite formation and reduces the later corrosion rates. Substrate such as stainless steel modified with an aligned graphene layer^5^, shows good lattice matching with Zn, which induces epitaxial deposition of Zn along the (002) planes, achieving ultra-long cycling life. Recently, fields generated by rotating disc electrodes^19^ are reported to promote the crystallographic reorientation of Zn to be grow parallel to the substrate, and the reversibility of Zn deposition/stripping is greatly increased. Therefore, correlating the crystallography and morphology to deeply understand and regulate the electrodeposition behavior of Zn is of great significance for developing long-life Zn batteries. However, there is an obvious lacks of fundamental elucidation of the mechanism controlling planar Zn deposition. Furthermore, the surface stability of inorganic crystals has long been thought to be dominated by their surface energy^31–33^. From the perspective of crystal growth, controlling the surface energy of Zn crystal planes offers exciting opportunities to realize planar zinc deposition.

**Fig. 1:**
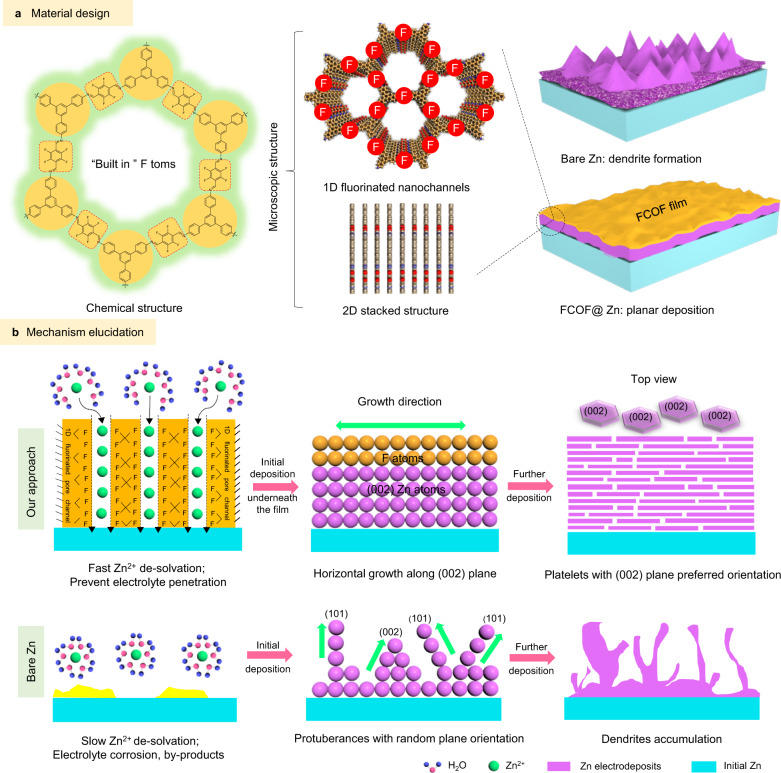
The FCOF structure design and stabilizing mechanism elucidation. **a** The physicochemical structure of the FCOF film, showing suppression of dendrites. **b** Mechanism comparison of the deposition processes for FCOF@Zn and bare Zn surfaces.

Here, by using two-dimensional (2D) covalent organic frameworks (COF) as a multi-functional platform (Fig. 1a), we develop a mechanically strong, ultra-thin, porous, and fluorinated COF (FCOF) film as a protective layer on Zn anode surfaces (FCOF@Zn). Compared with alterative protective layer materials, FCOF film is advantageous. This is because: i) From the perspective of regulation of the surface energy of Zn crystal, it introduces numerous F atoms into the FCOF film. The electronegative F atoms exhibit strong interaction with the underlying Zn atoms, leading to a lower surface energy for Zn (002) planes compared with that of conventional Zn (101) planes. Consequently, Zn deposits show platelet morphology with preferred orientation along the (002) plane, with the platelets arranged parallel to each other to give a planar Zn deposition morphology; ii) The FCOF film is continuous and dense, and has strong adhesion with Zn, that remains intact on the surface of Zn to provide durable protection; iii) The 2D stacking and covalent bonding makes the film excellent mechanical properties. The robust film possesses an elastic modulus >30 GPa, which buffers volume expansion of Zn during cycling; and, iv) The FCOF film is ultra-light in mass, thin (100 nm) and is precisely regulated on a nanometer-scale without impacting mass or volume energy density of Zn anodes. As a result, the FCOF@Zn anodes show prolonged cycle life and better reversibility in a large current density range (5–80 mA cm^−2^). The assembled full cells paired with manganese dioxide (MnO_2_) cathodes show a stable cycle life for over 250 cycles under practical condition of lean electrolyte, high areal capacity cathode and limited Zn excess.

## Results

### Synthesis and characterization of the FCOF film

**Fig. 2:**
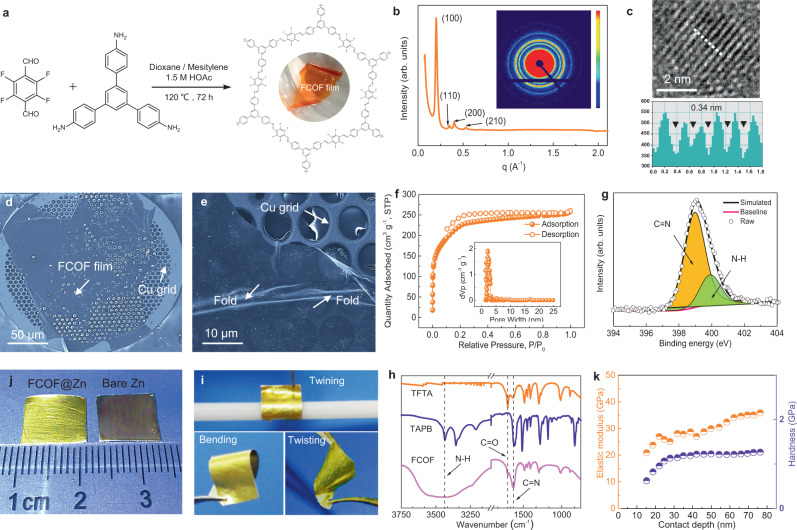
The morphology and structure characterization of the FCOF film. **a** Synthesis procedure of the FCOF film. **b** WAXS result and its integrated curve. **c** HRTEM image and measurement of *d-*spacing. **d**, **e** FESEM images of the FCOF film. **f** BET measurement and pore size distribution. **g** XPS result for N1*s* in the FCOF film. **i**, **j** Pictures of typical FCOF@Zn and typical bare Zn. **h** FTIR result for the monomers and the FCOF film. **k** Nano-indentation measurement result for FCOF film.

The imine-linked FCOF thin films are prepared through a solvothermal procedure (Fig. 2a). In a typical process, two monomers (2, 3, 5, 6-tetrafluoroterephthaldehyde (TFTA) and 1, 3, 5-tris (4-aminophenyl) benzene (TAPB)), are dissolved in a dioxane/mesitylene (D/M) mixture and condensed in a solvothermal tube, using acetic acid as the catalyst. As a new COF film material, the production cost of FCOF is comparable to those for reported microporous metal-organic frameworks (MOFs) or COF material (Supplementary Table 1). Optimization of the synthesis pathways and cost of COFs can be used to reduce overall cost for practical commercialization (details in Supplementary Table 1). To obtain a highly crystalline and continuous FCOF film, reaction conditions i.e., the proportion of solvent mixture, the concentration of catalyst and the reaction time needs to be controlled (Supplementary Figs. 1–4). When the ratio of D:M solvent in the mixtures is optimized to 9:1 (v/v) with 1.5 M acetic acid catalyst, continuous bright orange FCOF films without any insoluble COF particulates are uniformly attached to the tube inner wall, indicating the FCOF films is successfully achieved (Supplementary Fig. 1). After soaking in pure water overnight, the free-standing FCOF films detach from the tube wall due to surface tension (Supplementary Fig. 5). The growth mechanism of FCOF film during solvothermal processes is mainly attributed to the fusion of numerous nanospheres that are formed during a co-condensation reaction (Supplementary Fig. 2), as revealed by the time-dependent morphology evolution (Supplementary Fig. 4). In addition, such uniform FCOF films can grow on various substrates such as copper (Cu), silicon, stainless steel foil/grids, nickel, and titanium foils by placing the targeted substrates in the reaction solutions, as shown in Supplementary Fig. 6. This is beneficial for structural and property characterization after being transferred to various substrates during subsequent post-processing.

The crystal structure of the FCOF film is identified by two-dimensional wide-angle X-ray scattering (2D WAXS) measurement (Fig. 2b). From the integrated WAXS curves, the peaks at *q* = 0.20, 0.35, 0.40, 0.53 A^−1^ correspond to the planes (100), (110), (200) and (210), respectively, consistent with a previous report^34^, confirming the high degree of crystallinity of the as-prepared film. Findings from theoretical simulation and Pawley refinement confirm that the 2D stacked structure in FCOF is an AA stacking mode (detailed analysis in Supplementary Fig. 7). High-resolution transmission electron microscopy (HRTEM) images clearly show the lattice fringes of the FCOF film at a spacing of ~0.34 nm (Fig. 2c), which represents the π-π stacking distance. Field emission scanning electron microscopy (FESEM) images show a smooth and defect-free film suspended on a copper grid (Fig. 2d), and the folds at the edges also reflect the flexibility of the film to a certain extent (Fig. 2e). Brunauer–Emmett–Teller (BET) measurement indicates the surface area of the film is as high as 723 m^2^ g^−1^, and the main pore size distribution is 2–3 nm in diameter (Fig. 2f), which is well consistent with the WAXS result. The chemical structure of the FCOF film is further confirmed. The emerging peak at 1614 cm^−1^ in FTIR spectra (Fig. 2h) is assigned to the newly formed C=N imine stretch vibrations. The peak intensity at 1705 cm^−1^ assigned to C=O stretching weakens in FCOF film, indicating the consumption of the aldehyde groups of TFTA monomers^34^. High-resolution X-ray photoelectron spectroscopy (XPS) of the N1*s* spectra (Fig. 2g) shows that the weak peak at 399.89 eV arises from the N–H bonds^35^, revealing the small amount of residues of the amino groups^34^, consistent with the FTIR result (N–H peak at ~3400 cm^−1^). The high intensity of the peak at 398.96 eV in the N1*s* spectra (Fig. 2g) further confirm the formation of C=N (imine) bonds in the FCOF film^35^.

The F atoms are the crucial elements within the FCOF films for achieving high-performance Zn anodes. The element mapping obtained from energy dispersive X-ray spectroscopy (EDX) indicates that F is evenly distributed in the film (Supplementary Fig. 8). The F content is estimated to be 8.25 atomic %, in according to the XPS result (Supplementary Fig. 9). In addition, the thickness of the film is adjustable by means of controlling the concentration of monomers. As determined by AFM analysis, the FCOF films have thicknesses of ~100, ~300, and ~500 nm (Supplementary Fig. 10). It is worth mentioning that due to the formation mechanism of the films, a minimum limitation of thickness exists for preparing the FCOF film (Supplementary Fig. 11). To suppress the side reactions and retard the Zn dendrites, a reliable film with a thickness of about 100 nm is optimal to conduct the subsequent characterization. The FCOF@Zn anode is fabricated via a pulling method in acetone solvent using Zn foil as substrate. After drying, the FCOF film tightly adheres the surface of the Zn foil and does not detach even under rolling, bending or unfolding of the Zn (Fig. 2i, j). In addition, as determined by nano-indentation measurements (Fig. 2k), the high quality two-dimensional FCOF crystalline films show a remarkable elastic modulus exceeding 30 GPa and an average hardness of over 1.2 GPa, which is an order of magnitude higher than a recently reported TiO_2_ and polyvinylidene difluoride (PVDF) hybrid matrix (2.67 GPa)^36^. The good mechanical strength is greatly beneficial to buffer volume expansion and retard dendrite propagation during the dissolution/deposition of Zn anodes.

### Ion de-solvation promotion and transport acceleration in the FCOF film

Good Zn^2+^ conductivity through a protective layer is highly desired for Zn anodes. To experimentally determine the ion transport behavior of the FCOF films, the ionic conduction is calculated based on electrochemical impedance spectroscopy (EIS) results (Supplementary Figs. 12 and 13). As is shown in Supplementary Fig. 12, the FCOF films exhibits a greater Zn^2+^ transference number ZTN of 0.75 compared with conventional glass fiber separators of 0.22. The ion conductivity of the ~100 nm FCOF film coated on glass fiber separator is computed to be 24.19 mS cm^−1^, that is, 1.7 times greater than that for bare glass fiber separator of 14.12 mS cm^−1^. These results confirm that FCOF accelerates Zn^2+^ transport and therefore boosts ionic conductivity^37,38^. According to the equivalent circuit fitting results (Supplementary Fig. 13), Zn anodes coated by the FCOF films with different thicknesses reveal lower charge transfer resistance (*R*_ct_) than bare Zn. In particular, the *R*_ct_ of Zn anodes coated by the 100 nm thick FCOF film is lowest (90 Ω), about half that of bare Zn (180 Ω). Apparently, the Zn^2+^ transport is increased by the fluorinated 1D nanochannels. This is mainly due to the F atoms surrounded within the nanopores that endow the film a strong hydrophobic effect. When hydrated Zn^2+^ transport through fluorinated nanochannels is driven by electric field force, water molecules coordinated with Zn^2+^ will be repelled because of the strong hydrophobicity of F element in the FCOF film covering the Zn metal. Consequently, a large portion of water molecules is retarded and not able to penetrate the fluorinated nanochannels. The de-solvation effect of Zn^2+^ is therefore significantly promoted by F element. To confirm the ability of FCOF film to promote de-solvation, a study of the activation energy (*E*_a_) is made, *E*_a_ represents the de-solvation barrier to Zn^2+^ transport. A computation for *E*_a_ based on temperature-dependent EIS is performed (Supplementary Fig. 14). To determine *E*_a_, temperature-dependent EIS curves for FCOF@Zn and bare Zn anodes are determined. The EIS are fitted with an equivalent circuit, and *R*_ct_ at different temperatures is obtained. It is found that *R*_ct_ for FCOF@Zn is several orders of magnitude lower than that for bare Zn when evolving at different temperatures (Supplementary Fig. 14a, c). According to the equation:

1 $${{{{{\mathrm{ln}}}}}}({R}_{{{{{{\rm{ct}}}}}}}^{-1})=\,{{{{{\mathrm{ln}}}}}}\,A-{E}_{{{{{{\mathrm{a}}}}}}}/{{{{{\rm{RT}}}}}},$$

where *R*_ct_, *A*, *E*_a_, *R*, and *T* represent the charge transfer resistance, pre-exponential factor, activation energy, molar gas constant, and absolute temperature. The computed activation energy (*E*_a_) for FCOF@Zn is 14.5 kJ mol^−1^, whilst that for bare Zn is significantly greater at 32.2 kJ mol^−1^. The lower activation energy for FCOF@Zn over bare Zn strongly suggests that the FCOF layer ensures fast de-solvation of Zn^2+^ and facilitates fast ion-transference. Studies have demonstrated that COFs are an excellent ionic conductor^38,39^. The inherent ordered structures provide regular ionic coordination sites to facilitate ions to hop along the 1D aligned nanochannels. This highly boosts ionic conductivity. To study Zn^2+^ transport in FCOF, the transport sites for Zn^2+^ on the COF skeleton are determined theoretically. The charge density distribution of the chemical structural unit in FCOF is computed. As is shown in Supplementary Fig. 15a, the significant number of F atoms within FCOF film (four (4) F element per unit) exhibit a strong local, negative charge concentration distribution. The pore channels of COF with strong negative charge coordination sites have been confirmed to boost fast ion movement^38,39^. Because of electrostatic attraction, the positively charged Zn^2+^ hops around the F atom sites and transports along the 1D aligned channels during charge/discharge (Supplementary Fig. 15b, c). This shortens the transportation pathways, and Zn^2+^ transport is boosted.

### Corrosion resistance property of the FCOF film

The corrosion resistance of the FCOF film on the Zn surface is investigated in aqueous electrolyte (2M ZnSO_4_). The impedance of the FCOF@Zn symmetric cells increases from 180 to 600 Ω within 8 h (h) after cell assembly (Supplementary Fig. 16a). For bare Zn symmetric cells, on the contrary, the impedance increases dramatically from 200 to over 10,000 Ω after 8 h (Supplementary Fig. 16b). The increase in impedance implies that the continuous corrosion of Zn by the electrolyte results in large amounts of by-products deposited on the surface. Time-dependent XRD patterns (Supplementary Fig. 16c) show a peak at around 8° appears, corresponding to the by-product species Zn_4_SO_4_(OH)_6_·5H_2_O (JCPDS# 39-0688)^11^. When plain Zn anodes are immersed in aqueous electrolyte for 48 h, the peak intensity of by-products increases sharply. Much less irreversible by-product is accumulated on the surface of FCOF@Zn anodes during the same time duration. To evaluate corrosion resistance of the FCOF film, potentiodynamic and open-circuit, tests are conducted using a home-made test apparatus (Supplementary Figs. 17 and 18). The time-dependent potentiodynamic curves for FCOF@Zn and bare Zn in 2 M ZnSO_4_ electrolyte are shown in Supplementary Fig. 17. Following soaking in electrolyte for 2 h, the *E*_corr_ for FCOF@Zn is −1.057 V, a value slightly greater than that for bare Zn of −1.063 V. Additionally, the corrosion rate is proportional to computed corrosion current. The corrosion current (*I*_corr_) decreased from 2.5 × 10^−3^ A cm^−2^ for bare Zn to 4.1 × 10^−5^ A cm^−2^ for FCOF@Zn. Despite 8 days of electrolyte immersion, FCOF@Zn continued to exhibit a greater corrosion potential (*E*_corr_) of −1.063 V and a lower corrosion current (*I*_corr_) of 4.8 × 10^−4^ A cm^−2^, compared with bare Zn of, respectively, −1.068 V and 1.2 × 10^−3^ A cm^−2^. The open circuit potential (OCP) for both electrodes is shown in Supplementary Fig. 18. The corrosion potential for bare Zn in 2 M ZnSO_4_ remained stable at ~ −1.042 V vs. SCE, whilst that for FCOF@Zn is ~ −1.020 V in the initial stage, and remained greater at −1.032 V than that for bare Zn of −1.041 V, despite 5000 s. It is reliably concluded therefore that the corrosion resistance for Zn anode is significantly improved by FCOF film surface protection.

### Horizontal parallel Zn platelet deposition enabled by the FCOF film

**Fig. 3:**
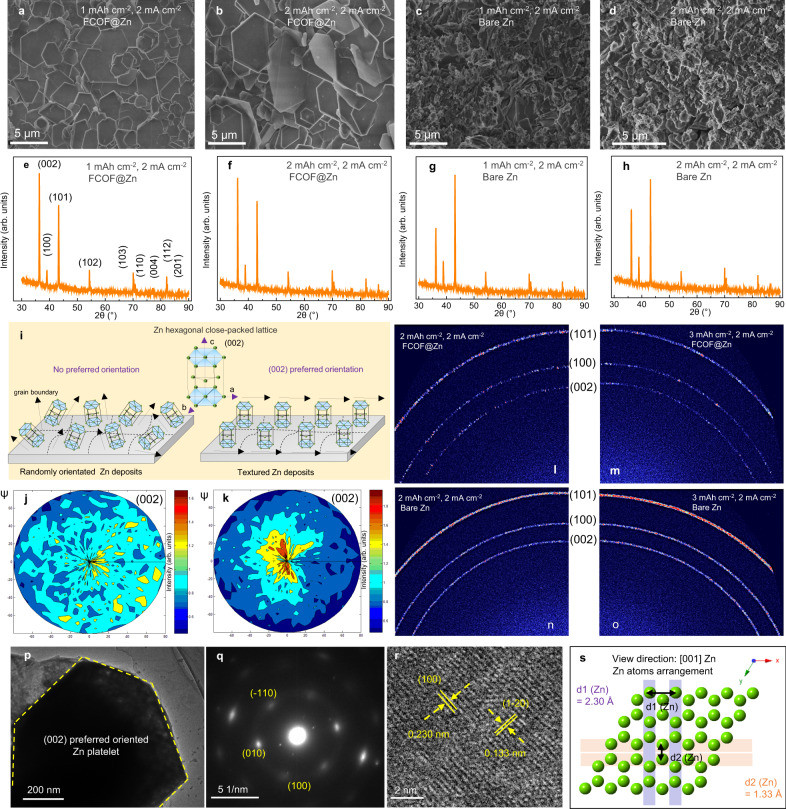
Morphology, crystallography and microstructure characterization of Zn electrodeposits. FESEM images of Zn deposits **a**, **b** underneath a FCOF film and **c**, **d** on bare Ti. XRD results for the Zn deposits **e**, **f** underneath the FCOF film and **g**, **h** on bare Ti. **i** Schematic illustration of preferred orientations of Zn crystal plane. (002) plane pole figures of the Zn deposits **j** on bare Ti and **k** underneath FCOF film. The WAXS results of Zn deposits **l**, **m** underneath FCOF film and **n**, **o** on bare Ti. **p**–**r** HRTEM images and SAED patterns of the Zn platelet. **s** Theoretical atomic model of Zn along the [001] direction.

In addition to the fast ion conduction and suppression of side reactions features, the morphology and texture of Zn deposits has been proven to have large impact on the cycling life of Zn batteries. Attaining an even planar deposition can ensure the batteries running for a prolonged time without short circuiting. An electronic resistance property is important in Zn deposition underneath the FCOF film. From the polarization I–V curve, the 100 nm FCOF film exhibits an electronic resistance of 3 × 10^4^ Ω cm (Supplementary Fig. 29d). This is a value has been to be sufficient to enable Li deposition underneath the protective layer^40^. It is concluded the FCOF film therefore provides good electronic insulation on the surface of Zn, and establishes the needed potential gradient across the film to drive Zn^2+^ diffusion through the film. Additionally, the F-containing channels inside the film provide a rapid de-solvation environment and 1D ion diffusion pathways (Supplementary Fig. 19) that allow Zn^2+^ to pass through the film and readily deposit on the Zn surface. To investigate the deposition morphology of Zn underneath the FCOF film, the Ti/Zn or FCOF@Ti cells are employed. As shown in Fig. 3a, b, the Zn deposits underneath the FCOF film exhibits platelet morphology and the platelets are stacked horizontally in response to a controlled capacity of 1 mAh cm^−2^. Meanwhile, for the bare Ti without FCOF film protection (Fig. 3c, d), disordered, distributed, and irregularly-shaped Zn dendrites are observed on the surface. When further increasing the used capacity to 2 mAh cm^−2^, similar consistent morphological characteristics of the two samples are still maintained. A comparison of deposition morphology is shown in Supplementary Figs. 20 and 21. It can be seen that Zn deposition on Zn surface underneath the FCOF film exhibits horizontally arranged platelet morphology. SEM images with low magnification confirm platelet deposition morphology over a range of tens-of-microns (Supplementary Figs. 20–22). This confirms that platelet electrodeposition facilitated by FCOF film is ubiquitous, and, importantly, is therefore scalable for Zn battery anodes. The XRD results reveal the intensity of (002) plane located at 2*θ* = 36.3° is highest for the Zn deposits underneath FCOF films (Fig. 3e, f), while the bare Zn deposits show (101) planes dominating the peak intensities (Fig. 3g, h). This change in the dominant peaks implies that the FCOF films on Zn anodes influence the preferred orientation of the Zn deposits. It is noted that the XRD peaks located at 8.5°, 16.8°, 20.6°, and 24.8° are observed for bare Zn deposits (Supplementary Fig. 23), and are attributed to Zn_4_SO_4_(OH)_6_ •5H_2_O (JCPDS NO: 39-0688) by-product. In contrast, these by-product signals are significantly weaker for Zn deposits under the FCOF film, strongly evidencing that the FCOF film provides protection for Zn, and inhibits accumulation of by-product. The orientation of the Zn deposits can also be quantified by calculating the texture coefficient^41^ (*T*_c_, Supplementary Fig. 24). The *T*_c_ (002) of Zn deposits underneath the FCOF film is 19.2, much higher than that of the deposits on bare Zn (11.5), verifying the preferential growth on the (002) plane of Zn modulated by an FCOF film. XRD patterns for Zn deposits following the 30th and 80th cycles are shown in Supplementary Fig. 25. Results highlight that the Zn deposits underneath the FCOF film maintain preferred (002) plane orientation (Supplementary Fig. 25b), whilst bare Zn deposits have a (101) crystal-plane orientation (Supplementary Fig. 25a). Significantly, for the FCOF@Zn electrode (Supplementary Fig. 25c), *T*_c_ for the (002) plane is stable with increase in cycle number (1st cycle: 19.2; 30th cycle: 18.42; 80th cycle: 18.31), however is higher than that for bare Zn (1st cycle: 11.5; 30th cycle: 11.12; 80th cycle: 10.34). These evidences validate that the preferred (002) plane orientation is maintained, despite long cycling.

X-ray diffraction pole figures are used to further identify the texture information of Zn deposits. The (002) pole figure (Fig. 3k) of Zn underneath FCOF films shows a sharp intensity concentration around ψ = 0–20°, indicating that the Zn platelets have a preferred textured based on (002) planes, and are nearly paralleled to the electrode substrate^24,42^ (Fig. 3i). In contrast, the random distributed of bare Zn deposits leads to a broad distribution of grain orientations, and the corresponding (002) pole figure (Fig. 3j) shows almost uniform distribution of diffraction intensity along the radial direction, indicating its random (non-preferential) texture. In addition, the 2D WAXS patterns of deposited Zn underneath FCOF film show some strong, discrete diffraction spots in the ring plane (Fig. 3l, m), while for bare Zn deposits, the WAXS results are continuous diffraction rings (Fig. 3n, o). This indicates that the bare Zn deposits are polycrystalline and randomly oriented, whereas the Zn grain size influenced by FCOF films is larger and more oriented^5^. The structure of the Zn platelets is characterized by HRTEM and selected area electron diffraction (SAED). As shown in Fig. 3p, q and Supplementary Fig. 26, the diffraction patterns of the SAED results can be indexed into diffraction spots of the [001] zone. The HRTEM image of Fig. 3r further shows two *d*-spacings of 0.230 and 0.133 nm with an interfacial angle of 90°, corresponding to the (100) and (1–20) planes, respectively. The HRTEM result is in accord with the indexed SAED diffraction spots. To further verify the indexing results, an atomic arrangement model of Zn along the (001) direction is simulated, as shown in Fig. 3s. Obviously, the indexed result matches well with the theoretical crystal model. According to the above results, we conclude that the exposed hexagonal planes of the Zn platelet are predominately (002) planes.

### Performance evaluation of the high-rate and long-life zinc anode

**Fig. 4:**
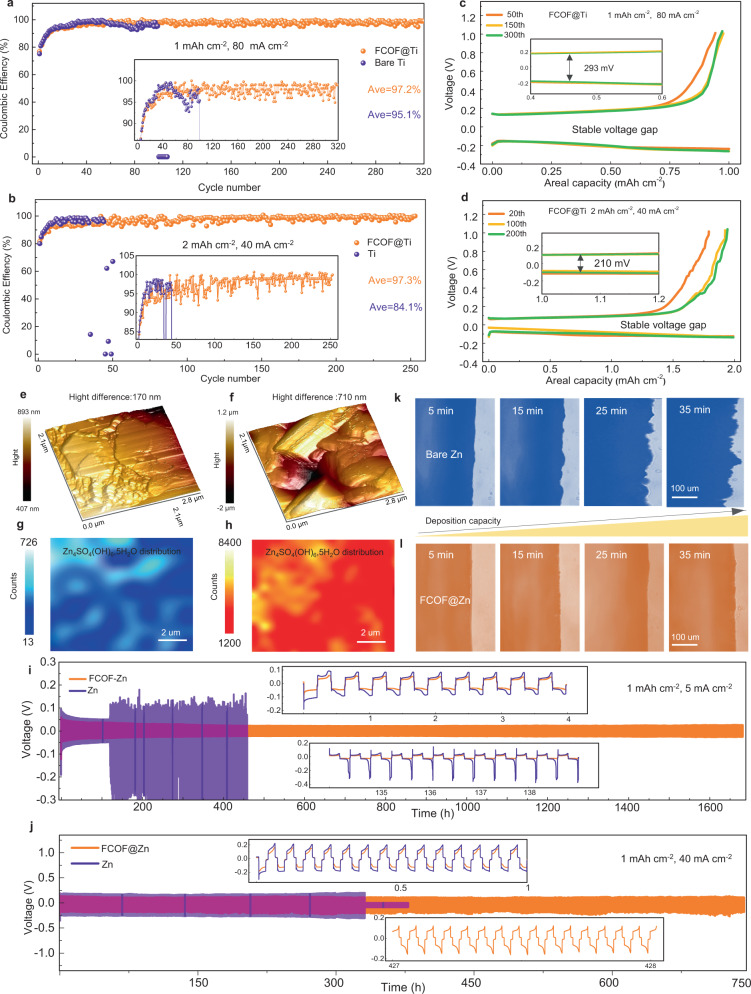
The electrochemical performance of Zn anodes. CE of Zn plating/stripping on FCOF film-coated Ti and bare Ti at **a** 1 mAh cm^−2^, 80 mA cm^−2^ and **b** 2 mAh cm^−2^, 40 mA cm^−2^. **c**, **d** The corresponding voltage profiles at various cycles on FCOF film-coated Ti. The insets are enlarged voltage profiles. AFM 3D height imaging and 2D Raman mapping on the surfaces of Zn deposits after 200 cycles plating/stripping (1 mAh cm^−2^, 5 mA cm^−2^), **e**, **g** on FCOF film-coated Ti and **f**, **h** bare Ti. Cycling performance of Zn symmetric cells with or without FCOF film protection at **i** 1 mAh cm^−2^, 5 mA cm^−2^, and **j** 1 mAh cm^−2^, 40 mA cm^−2^. The insets are initial and selected voltage-time profiles. In situ optical microscopy studies on Zn deposition behaviors. **k** Bare Zn, **l** FCOF@Zn.

The planar Zn deposition morphology, fast Zn^2+^ transport, and corrosion resistance properties enabled by the FCOF film are expected to greatly improve the electrochemical performance of Zn anodes. The reversibility of Zn anodes can be measured by a procedure that wherein a specific amount of Zn is plated on the substrate and then stripped away. Coulombic efficiency (CE) is an important index to evaluate such reversibility. The CE using the half cells in FCOF@Ti/Zn and Ti/Zn configurations is measured. At a moderate current density (1 mAh cm^−2^, 5 mA cm^−2^, Supplementary Fig. 27a–c), the FCOF@Ti/Zn cells produced CE values of ~98.4% on average, with stability over 480 cycles. By contrast, the Ti/Zn with no FCOF cells ran for only 30 cycles, and their CE is around ~95.1%. When the current density is increased to an ultrahigh current density of 80 mA cm^−2^ (Fig. 4a), the FCOF@Ti/Zn cells still exhibit a high CE of approaching 97.2% on average within for 320 cycles, whereas the CE of the Ti/Zn cells decreases rapidly after 95 cycles. When further increasing the capacity to 2 mAh cm^−2^ at a current density of 40 mA cm^−2^, the FCOF@Ti/Zn cells show CE of 97.3% for over 250 cycles, much higher than that of the Ti/Zn cells (~35 cycles, 84.1%). Remarkably, as evidenced from the AFM height and phase imaging (Fig. 3e, f and Supplementary Fig. 28a and c), the horizontally arranged platelet morphology of the Zn deposits underneath FCOF remains well after 100 cycles during Zn plating/stripping processes (1 mAh cm^−2^, 5 mA cm^−2^). The average height difference (along *X* and *Y* axis) is only 170 nm (Supplementary Fig. 28e), indicating the surface of the Zn deposits underneath FCOF is very flat and homogeneous. However, the Zn deposits on bare Ti after 100 cycles shows fluctuating and rough patterns with a much higher height difference of 710 nm (Supplementary Fig. 28b, d, and f). During the Zn plating/stripping process, the H^+^ from the decomposition of water will receive electrons and then evolve H_2_, which could induce an increase of OH^−^. The generated OH^−^ will react with Zn^2+^, SO_4_^2−^, and H_2_O to form by-products such as Zn(OH)_2_ or Zn_4_SO_4_(OH)_6_·nH_2_O on Zn surface^16^. Raman spectroscopy is carried out to reveal the components on Zn deposits surfaces after cycling (100 cycles at 1 mAh cm^−2^, 5 mA cm^−2^). Sharp peaks at 1152, 1110, 1011, and 967 cm^−1^ are observed on the Zn deposits on bare Ti (Supplementary Fig. 29a), which implies that the by-product should be the Zn_4_SO_4_(OH)_6_·5H_2_O^43^. In contrast, the peaks of the Zn deposits underneath FCOF are not obvious and their intensity is much lower (Supplementary Fig. 29b), indicating less by-product accumulation on its surface. Raman mapping (8 × 10 μm area, Fig. 4g) of the dominated peak at 967 cm^−1^ reveals that the counts variation for the Zn deposits underneath FCOF is within 13–726, which is one to two orders of magnitude smaller than for Zn deposits on bare Ti (Fig. 4h, counts: 1200–8400). It has been reported that the Zn deposits with high percentage of (002) planes parallel to the substrate could provide higher corrosion resistance than other planes^44^. Combined with the F endowed hydrophobic properties, the water-related side reactions could be largely suppressed in the FCOF@Ti cells. Moreover, the electrochemical stability of the FCOF film is evaluated using CV measurements (Supplementary Fig. 30). No excess decomposition current is apparent. It is concluded therefore that the FCOF film is stable and did not react with Zn, or electrolyte, and is resistant to parasitic-chemical reactions during cycling. In addition, the bare Ti is not adequate for regulating the zinc deposition behavior or suppressing by-products accumulation, causing elevated voltage hysteresis or short circuiting of the batteries, as evidenced by the voltage fluctuation in Ti/Zn cells during cycling (Supplementary Fig. 27c–e). Whereas the voltage files of FCOF@Ti/Zn remain stable at various levels of current density (Supplementary Fig. 27a and Fig. 4c, d). Meanwhile, FCOF@Ti/Zn cells display long-term stability of the Zn plating/stripping process even at ultrahigh current density up to 80 mA cm^−2^, larger than that of most previous studies (Supplementary Table 2).

To evaluate the stability of the Zn anodes, the FCOF@Zn symmetric cells show prolonged cycle life for over 1700 h at 1 mAh cm^−2^ and 5 mA cm^−2^, which is nearly 13 times the performance of the bare Zn anodes (Fig. 4i). The FCOF@Zn symmetric cells show lower voltage hysteresis (FCOF@Zn: 60 mV vs. bare Zn: 80 mV), which we mainly attribute to the enhanced Zn^2+^ transport within the 1D fluorinated nanochannels. Under elevated current densities of 8 and 40 mA cm^−2^ (Supplementary Fig. 27e and Fig. 4j), the FCOF@Zn symmetric cells could sustain repeated deposition/dissolution processes without obvious significant fluctuations in the voltage-time curves. However, the bare Zn symmetric cells suffer short-circuits after a few limited cycles. The excellent performance of FCOF@Zn symmetric cells at ultrahigh current density (40 mA cm^−2^) is also far superior to most previous reported values (below 10 mA cm^−2^, Supplementary Table 3). Under higher cycling capacity conditions (Supplementary Fig. 31), including 2 mAh cm^−2^, and 3 mAh cm^−2^, FCOF@Zn maintains a more stable cycling performance than for bare Zn. The highly stable electrochemical performance of the FCOF@Zn anodes indicates that dendrite formation is largely suppressed. To identify the suppression of dendrite growth in FCOF@Zn anode, transparent home-made Zn/Zn symmetric cells with or without FCOF are assembled to realize in situ monitoring of the Zn deposition process using an optical microscope. Zn deposition is performed under a current density of 20 mA cm^−2^ for 35 min. As shown in Fig. 4k, after an initial 5 min of deposition, nonuniform Zn morphology with some protuberances appears on the bare Zn surfaces. These protuberances remain and grew into needle-like dendrites in the following deposition process. In contrast, the deposition on FCOF@Zn is smooth as evidenced in Fig. 4l. No obvious Zn dendrites are observed, even after 35 min deposition. The microscopic morphologies of the Zn anodes after cycling at 1 mAh cm^−2^and 5 mAcm^−2^, for 500 h are also investigated. As shown in Supplementary Fig. 32, the FCOF@Zn anodes show that dendrite-free morphology and parallel platelet-morphology is consistently maintained. In contrast, protuberant Zn dendrites are found randomly distributed on bare Zn surfaces. Moreover, the HRTEM and FTIR results (Supplementary Fig. 33) show the FCOF films maintain good crystallinity and chemical structure stability after cycling. Consequently, it can be concluded that the multifunctional F nanochannels greatly improve the Zn^2+^ kinetics and deposition morphology, which results in high-rate and long life FCOF@Zn anodes.

### Full cell performance and flexible device demonstration

**Fig. 5:**
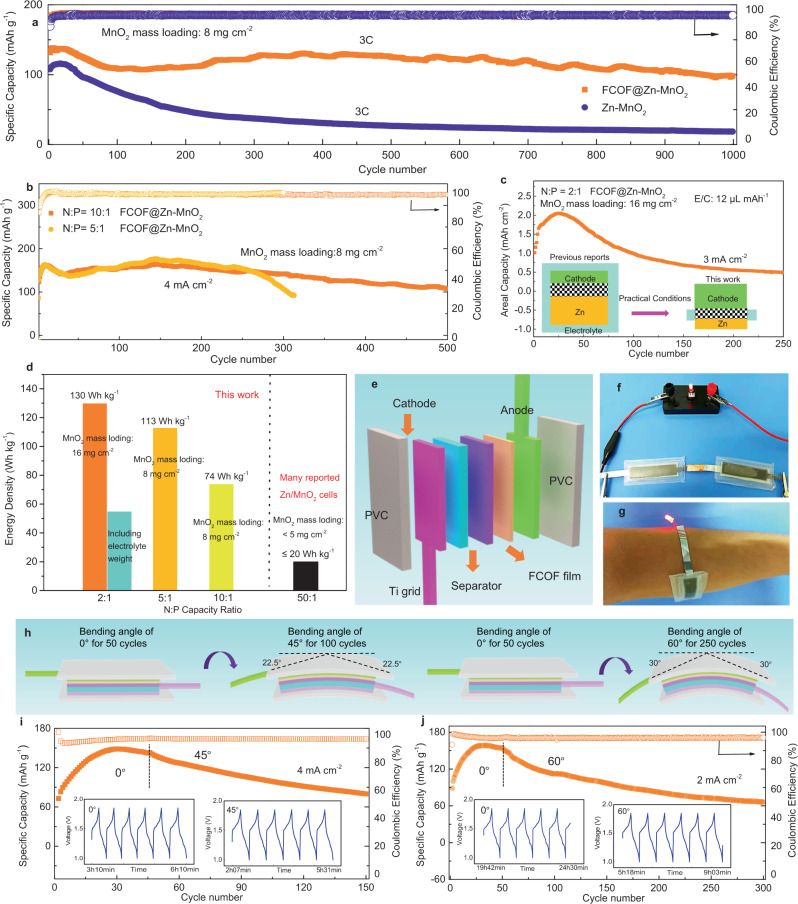
The full cell performance and flexible device demonstration. **a** Cycling performance at current density of 3 C. **b** Cycling performance at *N*:*P* capacity ratio conditions of 10:1 and 5:1. **c** Cycling performance at low *N*:*P* capacity ratio of 2:1 with controlled electrolyte addition of 12 μL mAh^−1^ **d** Dependence of cell-level energy density on *N*/*P* ratio in Zn/MnO_2_ full cell. The gravimetrical energy density is calculated based on the total mass of the Zn anode and the MnO_2_ cathode. **e** Assembly schematic illustration of the flexible transparent battery. **f**, **g** Pictures of the battery acting as a source of energy to power a LED. **h**–**j** Cycling performance of the flexible transparent battery under different bending angles. The insets are selected voltage-time profiles.

We next evaluate the electrochemical performance of full cells in which FCOF@Zn or Zn anodes are paired with high mass-loading (~8 mg cm^−2^) manganese dioxide (MnO_2_) cathodes. For the FCOF@Zn/MnO_2_cells, cyclic voltammetry curve (CV) curves demonstrate a larger current density at 0.1 mV s^−1^ and a smaller voltage gap between typical redox peaks than in Zn/MnO_2_ cells (Supplementary Fig. 34a). This implies that the FCOF@Zn/MnO_2_ cells possess higher specific capacity and better charge transfer capability^6^. EIS results further confirm that, the impedance of the FCOF@Zn/MnO_2_ cells (~100 Ω) is lower than that of Zn/MnO_2_ cells (Supplementary Fig. 34b). Therefore, at a current density of 3 C, the FCOF@Zn/MnO_2_ cells reveal a high initial reversible specific capacity of 130 mAh g^−1^, while the Zn/MnO_2_ cells attain only 120 mAh g^−1^ (Supplementary Fig. 34c). The FCOF films clearly endow stable cycling of the Zn anodes, and retain a capacity of ~92% and stable charge/discharge curves after 1000 cycles (Fig. 5a and Supplementary Fig. 34d, e). This is nearly four times higher than the Zn/MnO_2_ cells (capacity retention: 20%). Reducing the capacity ratio of the negative electrode to the positive electrode (*N*/*P*) during full cell operation is a key parameter to achieve high energy density^14,30,45^. In previous studies, many systems chose to use thick zinc foil (≥100 µm) paired with low mass loading cathodes to assemble full cells, the *N*/*P* reported in these studies is typically higher than 50, which is not beneficial for achieving high energy density. In our case, the excellent performance of the FCOF@Zn anodes allows us to further evaluate the cycle performance of full cells under harsh conditions. Using thin FCOF film-protected Zn plates as anodes (the thin Zn plates is rolled to desired thickness to satisfy the required *N*/*P* condition), FCOF@Zn/MnO_2_ cells with *N*/*P* = 10:1 and *N*/*P* = 5:1 show stable specific capacity at current density of 4 mA cm^−2^ for over 300 and 200 cycles, respectively (Fig. 5b). The Zn platelet morphology after cycling and stable charge–discharge curves indicate the FCOF films enable great performance improvements in Zn anodes (Supplementary Fig. 35). To evaluate the electrochemical performance of the aqueous Zn batteries for commercial applications under practical conditions, lean electrolyte addition and high areal capacity cathode is needed (inset of Fig. 5c). To understand the practicality of FCOF@Zn anode, full cells with high mass loading MnO_2_ cathode (16 mg cm^−2^) and controlled electrolyte-to-capacity ratio (*E*/*C*, 12 μL mAh^−1^) is assembled. When the mass loading of MnO_2_ increases from 8 to 16 mg cm^−2^, the charge and discharge curves for FCOF@Zn-MnO_2_ full cell exhibit low polarization as shown in Supplementary Fig. 36a. The FCOF@Zn-MnO_2_ full cell (cathode loading, 16 mg cm^−2^, *N*/*P* = 2:1 and electrolyte addition, 12 μL mAh^−1^) is cycled at a current density of 3 mA cm^−2^. The charge/discharge curves determined from different cycles almost overlap (Supplementary Fig. 36b), highlighting high cycle stability. Additionally, the assembled full cell exhibits a significant capacity of 0.5 mA h cm^−2^ following 250 cycles. The gravimetric energy density of the cell is 130 Wh kg^−1^ (based on the total mass of the Zn anode and the MnO_2_ cathode), which is significantly increased (by approx. 6.5 times) compared with many reported Zn/MnO_2_ cells using low mass loading cathodes and thick Zn foils^30^ (Fig. 5d). It should be noted that the cell still delivers an energy density of 55 Wh kg^−1^ when including the electrolyte weight. Further optimization of other key components such as separator membrane and electrolyte may improve the energy density of the cell.

To further demonstrate the application prospects of the FCOF@Zn anodes for constructing realistic, smart, high-performance aqueous Zn batteries, we assemble a flexible transparent battery for device demonstration. Figure 5e and Supplementary Fig. 37 shows the structural schematic diagram of the transparent battery. The MnO_2_ cathode and FCOF@Zn anode are fixed to the flexible PVC substrate, and glass fiber is used as the separator. All layers are sandwiched and the battery is then assembled by thermal sealing. The cycling performance of the flexible battery under different bending conditions is shown in Fig. 5h–j. The EIS results (Supplementary Fig. 38) and the charge and discharge curves (insets of Fig. 5i, j) remain nearly unchanged at bending angles of 0°, 45° and 60°, respectively, indicating its good mechanical stability and flexibility. To create a more realistic scenario, a flexible FCOF@ Zn/MnO_2_ battery is used to power a wearable bracelet for lighting a light emitting diode (LED) indicator (Fig. 5f, g), showing its promising application in portable wearable electronic devices.

## Discussion

**Fig. 6:**
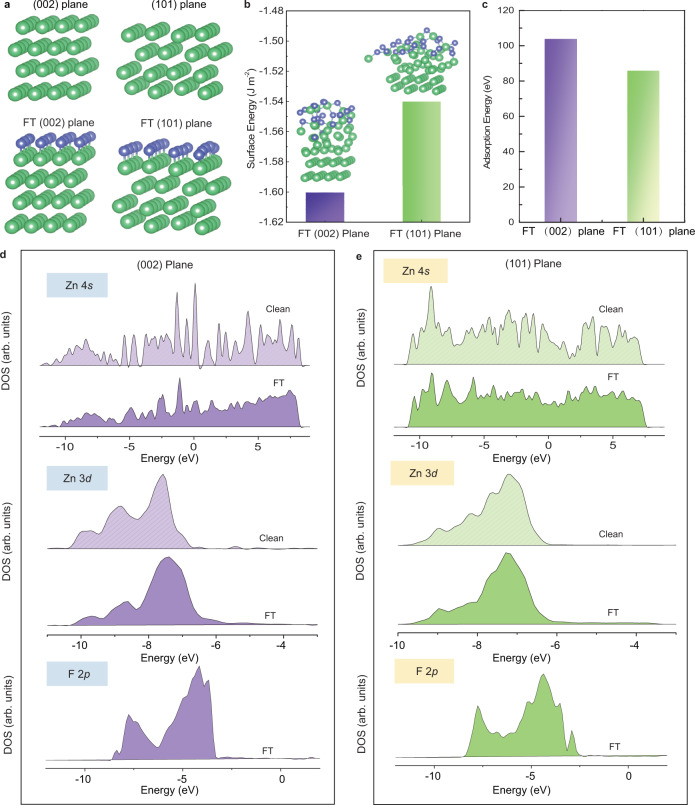
Theoretical simulation of FT-Zn (002) and FT-Zn (101). **a** Calculations models of F atom adsorbed on Zn (002) and Zn (101). Calculated **b** free energies and **c** adsorption energies of F atom on Zn (002) plane and Zn (101) plane. **d**, **e** Calculated projected density of states (PDOS) for clean Zn and FT-Zn systems.

The excellent electrochemical performance of the FCOF@Zn anodes can be mainly attributed to the planar deposition morphology, i.e., the predominantly parallel tessellated Zn platelets. This specific morphology seems to result from the tailored strongly-electronegative F atoms within the FCOF films, since the surface stability of inorganic crystals is largely governed by their surface chemistry. The preference of the exposed the crystal plane is closely related to the surface energy changes^31–33^. Previous studies indicated that the specific surface crystal planes in anatase surfaces are preferentially terminated by F atoms, inducing the anatase to expose specific crystal planes. Such a result is attributed to the surface energy decreases after the interaction between F and these crystal planes^31,46^. The F terminated (FT) plane with lowest the surface energy tends to stay exposed instead of its intrinsic thermodynamically stable plane. In our case, the uniformly distributed F atoms within FCOF film have a strong binding interaction with the Zn atoms, thereby regulating the relative surface energy value of the (101) and (002) planes and influencing the deposited morphology. To verify this, the surface energy and adsorption energy of the two crystal planes terminated by F atoms are studied by first-principles calculations (Fig. 6a). The calculated energy values clearly show that F atoms result in bonding to (002) planes that are more stable than (101) planes (Fig. 6b), and F atoms interact more strongly with the (002) planes (Fig. 6c), in accordance with the above XRD results. Moreover, with the formation of Zn–F interactions, the equilibrium positions of the Zn atoms on the surface of the crystal surface are clearly and obviously moved outward from where they would be without the presence of F atoms (insets of Fig. 6b). To further elucidate the stability mechanisms associated with F atoms, the electronic structures of clean surfaces and FT surfaces are investigated. The PDOS of clean and fluorinated surfaces are shown in Fig. 6d, e. For the clean surfaces, electrons contributed by Zn 4*s* delocalize in the range of the higher valence band (VB) and lower conduction band (CB). With the formation of Zn–F interaction, however, localized states of Zn 4*s* are observed, indicating that F atoms are prone to stabilize Zn atoms. To qualitatively determine the strength of the interaction between F atoms and Zn atoms and its impact on different crystal planes, the electron states of the Zn 3*d* and F 2*p* are analyzed. Due to the strong electron-withdrawing property of F atoms, the F 2*p* could accept electrons from the Zn 4*s*. As a result, the Zn 4*s* planes would partially lose electrons, forming quasi-stable bonds with the F species. Thereafter, the electrons from the Zn 3*d* may be excited toward the Fermi level. The displacement of Zn 3*d* electrons from the FT-(002) planes is more energetically-favorable than from FT-(101) planes. On the other hand, compared with the F 2*p* electron states of the in FT-(101) planes, the F 2*p* electron distribution in a FT-(002) plane is more localized. These results clearly suggest that F atoms interact more strongly with the (002) planes than with the (101) plane. The XPS data for the F 1*s* and Zn 2*p* at the FCOF-Zn interface also indicate the existence of strong F–Zn interaction^47–50^ (Supplementary Fig. 39). Therefore, the FT-(002) planes are more stable and thus preferentially form the outer surfaces. It seems the (101) surfaces are preferentially eroded in a stepped fashion during electro-stripping so that the exposed surface, which remains and subsequently builds up Zn deposits is a (002) surface.

To experimentally clarify the formation mechanism of Zn platelets, the evolution of the morphology and crystallography during the Zn deposition growth is systematically investigated. To compare growth behavior of Zn both underneath FCOF film and on bare Ti, the deposition capacity is controlled by setting the cut-off deposition time, 40 s, 2, 6, and 12 min, at a current density of 5 mA cm^−2^. Corresponding SEM images are shown as Supplementary Figs. 40 and 41. In contrast to Zn deposits on the Ti surface, in which the dendrites are randomly oriented (Supplementary Fig. 41), the Zn deposits underneath the FCOF film are in the form of platelets with parallel orientation (Supplementary Fig. 40). High-magnification SEM images of the different deposition stages reveal the evolution (Supplementary Fig. 40). At the initial heterogeneous nucleation stage (40 s), small-sized and horizontally-arranged Zn platelets are found to be evenly distributed underneath the FCOF film. With increased deposition capacity, the Zn platelets gradually grow horizontally, and following the entire Ti surface being fully occupied, the deposition enters the next homogeneous growth stage (6 min). Because the upper layer has been uniformly covered by F atoms, the newly deposited Zn continues to grow in the horizontal direction of the (002) crystal planes (12 min). The XRD results confirm this finding. From the initial heterogeneous nucleation stage to the final homogeneous growth stage, the XRD patterns underscore that the Zn deposits under the FCOF film maintain the strongest signal of the (002) planes (Supplementary Fig. 42a), whilst the bare Zn deposits shows the strongest (101) signal (Supplementary Fig. 42b). This confirms that the Zn deposits underneath the FCOF film grow along the preferred orientation of the (002) planes. Moreover, it is observed that the crystal plane signals in the WAXS spectra of the Zn deposits underneath the FCOF (Supplementary Fig. 43a) show discrete diffraction spots on the (002) ring, revealing the existence of (002)-textured single-crystal Zn deposits. The presence of the single-crystal signal underscores homogeneous deposition of Zn under the FCOF film. This means that the initial small-sized Zn platelets continue to grow in the direction of their initial orientation. In contrast, the WAXS spectra for the bare Zn deposits show a continuous, dispersive diffraction ring (Supplementary Fig. 43b), underscoring its polycrystalline and randomly oriented features.

Through experimental and first-principles calculations, we have determined that the F atoms in the FCOF film demonstrate the strongest interaction with the (002) crystal planes of Zn. The growth of (002) planes is most stable during the deposition process, and anisotropic growth of Zn along other planes is done in such a way that a (002) plane is formed, resulting in platelet-like Zn deposits. Given that the F atoms bonded within the FCOF film are arranged in parallel along the surfaces of the current collectors, which induces each Zn platelet to also be arranged in parallel. Furthermore, the F-containing porous nano channels endow good hydrophobic effects on the film, which is beneficial for de-solvation and facilitates fast transport of hydrated Zn^2+^, as well as reducing the corrosion of Zn by the aqueous electrolyte. The presence of F atoms and the “built-in nanochannels” in FCOF films seems to endow multiple advantages: the FCOF@Zn anodes exhibit excellent fast charging properties and cycle stability. The FCOF@Zn anodes can sustain over 320 cycles with excellent reversibility of ~97.2% and the symmetric cells exhibit long cycle life up to 750 h at an ultrahigh current density of 40 mA cm^−2^. This work provides novel design concepts for the realization of planar deposition and thus dendrite-free Zn anodes. The 2D COFs films are versatile platforms that show distinct advantages for constructing high-performance batteries, owing to their adjustable pore sizes, tailored functional groups, light weight, and structural stability through covalent bonding. The 2D COF rational design of the approach may also prove useful for other dendrite-free, long-life, and high-safety metal anode batteries, such as lithium, sodium, potassium, and magnesium. Importantly, the stabilizing mechanism proposed to suppress dendrite formation is not limited to FCOF, it might therefore reasonably lead to material designs for advanced separators and liquid/gel/solid electrolytes to achieve high energy density batteries.

## Methods

### Preparation of FCOF films and MnO_2_ cathodes

For the typical synthesis of FCOF film, a pyrex tube is charged with 3.75 mg (0.0182 mmol) 2, 3, 5, 6-tetrafluoroterephthalaldehyde (TFA), 4.40 mg (0.0125 mmol) 1,3,5-Tris(4-aminophenyl) benzene (TAPB), 1.8 mL dioxane (Dio) and 0.2 mL 1, 3, 5-trimethylbenzene (TMB). After sonication for 10 min, 0.1 mL HOAc (1.5 M) solution is added. After this, the tube is frozen at 77 K (liquid N_2_ bath) and degassed by three freeze-pump-thaw cycles and finally sealed under vacuum conditions. The sealed tube is heated at 120 °C for 3 days. For the postprocessing, the tube is opened, washed with acetone several times and then the tube is charged with deionized water overnight. The free-standing FCOF films easily detach from the tube wall by gently shaking the tube. The obtained FCOF films is immersed in acetone for 2 days to wash out any impurities. Finally, the as-prepared FCOF films is transferred to various substrates via a pulling method in acetone solvent. The thickness of the membrane/film can be also easily tuned by controlling the concentration of monomers. The MnO_2_ cathodes are synthesized using an aging method according to a previous report^51^. First, 5.07 g manganese sulfate monohydrate (MnSO_4_·H_2_O) is put in 100 mL deionized water, followed by ultrasound and stir to ensure dissolution (named Solution 1). Then 4.32 g sodium hydroxide (NaOH) and 20 mL hydrogen peroxide (H_2_O_2_, 30 wt%) are dissolved in 180 mL deionized water (named Solution 2). Solution 2 is then dropwise added into Solution 1 under vigorous stirring conditions. The mixed solution is stirred for 1 h and aged at 25 °C for 24 h. The precipitated product is centrifuged for three times, washed with deionized water, and dried to obtain MnO_2_.

### Structural and chemical characterizations

Wide-angle X-ray scattering (WAXS) measurement is conducted on a XenocsXeuss2.0 with 8 KeV Cu Kα radiation. X-ray diffraction (XRD) data is measured by a Bruker D8 Advance with Cu-Kα X-ray radiation (*λ* = 0.154056 nm), using an operating voltage of 40 kV and a 40 mA current. Fourier transform infrared (FTIR) spectra are collected on a ThermoFisher Nicolet 6700 spectrometer. N_2_ adsorption–desorption isotherms are measured at 77 K on a Micromeritics TriStar II 3020 volumetric adsorption analyzer after degassing in a vacuum at 120 °C overnight. Atomic force microscope (AFM) is performed on a NT-MDT NTEGRA Spectra II microscope. Field-emission scanning electron microscopy (FESEM) images are acquired from a Zeiss Gemini SEM500, equipped with an Aztec X-Max Extreme energy dispersive spectrometer (EDS). High-resolution transmission electron microscope (HRTEM) images are collected from a JEM-2010F transmission electron microscope. XPS measurements are carried out with a Thermo Scientific K-Alpha+ spectrometer under vacuum. Nano-indentation surface hardness measurements are conducted on a TI-950, NHT.

### Electrochemical measurements

Cycling tests for symmetric cells and Zn/Ti half cells of bare Zn or FCOF@Zn are conducted with 2 M ZnSO_4_ aqueous electrolyte. For cathode fabrication, the high mass loading MnO_2_ cathodes are prepared by mixing the active materials with carbon black and polytetrafluoroethylene (PTFE) in a mass ratio of 7:2:1. The mixture is compressed onto a Ti grid. The electrodes are dried at 80 °C under vacuum for 12 h and then punched into disks. All the electrochemical properties are tested by assembling 2016-coin cells with glass fiber separators. All Galvanostatic charge-discharge measurements are carried out on a battery testing instrument (Land CT2001A, Wuhan China) at different current densities. Electrochemical impedance spectroscopy (EIS) and cyclic voltammetry (CV) are performed by a CHI 660E electrochemical workstation. CV curves of full cells are recorded over the voltage range of 1–1.85 V. EIS is measured in a frequency range of 100 kHz to 0.1 Hz at open circuit potential and an amplitude of 5 mV. The fitting parameters of the equivalent circuit are analyzed by ZSimpWin software.

### Calculation method

The DFT calculations are carried out using the Vienna Ab-initio Simulation Package (VASP) with the frozen-core all-electron projector-augment-wave (PAW) method. The Perdew–Burke–Ernzerhof (PBE) form of the generalized gradient approximation (GGA) is adopted to describe the exchange and correlation potential. The cutoff energy for the plane-wave basis set is set to 500 eV. The Monkhorst-Pack k-point6 sampling is set to 3 × 3 × 1. The geometry optimizations are performed until the forces on each ion is reduced below 0.05 eV/Å. The vacuum slab models are used to calculate the adsorption of F atom on Zn (002) and (101) surfaces. These Zn surface slabs comprise four layers of Zn atoms, and a vacuum region of 20 Å above them is used to ensure the decoupling between neighboring systems. For the geometry optimization, the atoms in the 2-bottom layers of slab are fixed to their bulk positions.

The adsorption energy, ${E}_{{{{{{{\mathrm{ads}}}}}}}}$, is calculated using the expression:

2 $${{{E}}}_{{{{{{\rm{ads}}}}}}}={{{E}}}_{{{{{{\rm{surface}}}}}}}{+{{{{{\rm{16E}}}}}}}_{{{{{{\rm{F}}}}}}}-{{{E}}}_{{{{{{\rm{F}}}}}}+{{{{{\rm{surface}}}}}}}$$

where ${E}_{{{{{{{\mathrm{surface}}}}}}}}$ is the energy of the clean Zn surface, ${E}_{{{{{{\mathrm{F}}}}}}}$ represents the energy of the F atom, and ${E}_{{{{{{\mathrm{F}}}}}}+{{{{{{\mathrm{surface}}}}}}}}\,$ represents the total energy of the adsorbed F/Zn system.

The surface free energy ($\gamma$), is calculated using the expression based on a previous study^31^:

3 $${{{{{\rm{\gamma }}}}}}=\frac{{{{E}}}^{{{{{{\rm{slab}}}}}}}-{{{{{{\rm{NE}}}}}}}_{{{{{{\rm{Zn}}}}}}}^{{{{{{\rm{bulk}}}}}}}-{{{N}}}_{{{{{{\rm{F}}}}}}}{{{E}}}_{{{{{{\rm{F}}}}}}}}{{{{{{\rm{2A}}}}}}},$$

where, ${E}_{{{{{{{\mathrm{Zn}}}}}}}}^{{{{{{{\mathrm{bulk}}}}}}}}$ is the energy per unit of Zn, ${E}^{{{{{{{\mathrm{slab}}}}}}}}$ is the total energy of the slab, $N$ is the total number of unit Zn contained in the slab model, ${N}_{{{{{{\mathrm{X}}}}}}}$ is the number of adsorbed F atoms, ${E}_{{{{{{\mathrm{F}}}}}}}=\frac{1}{2}\,{E}_{{{{{{\mathrm{F-F}}}}}}}$− and ${E}_{{{{{{\mathrm{F-F}}}}}}}$ indicates the total energy of dimer F_2_.

## Supplementary information

Supplementary Information

## Data Availability

Source data are provided with this paper. Data supporting the findings of this study are available within the article and the associated Supplementary Information. The source data underlying Figs. 2–6 generated in this study is provided as a Source Data file. Additional data are available from the corresponding authors upon reasonable request. Source data are provided with this paper.

## Source data

Source Data
