# Supplementary material for: Horizontally arranged zinc platelet electrodeposits modulated by fluorinated covalent organic framework film for high-rate and durable aqueous zinc ion batteries
Source: Nat Commun. 2021 Nov 16;12:6606. doi: 10.1038/s41467-021-26947-9 (PMC8595410; doi:10.1038/s41467-021-26947-9)
Supplement: Supplementary file 1 — Supplementary Information [file 41467_2021_26947_MOESM1_ESM.pdf]

Supplementary Information for

Horizontally Arranged Zinc Platelet Electrodeposits Modulated by Fluorinated Covalent Organic Framework Film for High-Rate and Durable Aqueous Zinc Ion Batteries

Zedong Zhao[§1], *Rong Wang*[§1], *Chengxin Peng**[2, 4], *Wuji Chen*[1], *Tianqi Wu*[1], *Bo Hu*[1], *Weijun Weng*[1], *Ying Yao*[1], *Jiaxi Zeng*[1], *Zhihong Chen*[2], *Peiying Liu*[1], *Yicheng Liu*[1], *Guisheng Li*[2], *Jia Guo**[1], *Hongbin Lu**[1,5], and *Zaiping Guo**[3]

[1]*State Key Laboratory of Molecular Engineering of Polymers, Department of Macromolecular Science*

Fudan University, 2005 Songhu Road, Shanghai 200438, China;

[2]*School of Materials Science & Engineering, University of Shanghai for Science and Technology, Shanghai 200093, China*

[3]*Chemical Engineering & Advanced Materials, The University of Adelaide, Adelaide, SA 5005, Australia*

[4]*Key Laboratory of Advanced Energy Materials Chemistry (Ministry of Education), College of Chemistry, Nankai University, Tianjin 300071, China*

[5]*Yiwu Research Institute of Fudan University, Chengbei Road, Yiwu City, 322000 Zhejiang, China*

[§]*These authors contributed equally to this work*

Supplementary Figures

(a)

Raw material	Price list	Dosage	Cost
TAPB (-NH_2)	$61.7 /1g	3.75 mg/per tube	$0.22
TFTA (-CHO)	$ 77.0 /1g	4.4 mg/per tube	$0.34
Dio	$28.8 /100mL	1.8 mL/per tube	$0.51
Mes	$30.5 /100mL	0.2 mL/per tube	$0.06
Pyrex tube	$0.05 /per tube	One tube	$0.05
Total price（based on area）		$1600 /m² (7.5cm² film obtained)	
Total price（based on weight）		$483 /kg (the yields of film:30%)	

(b)

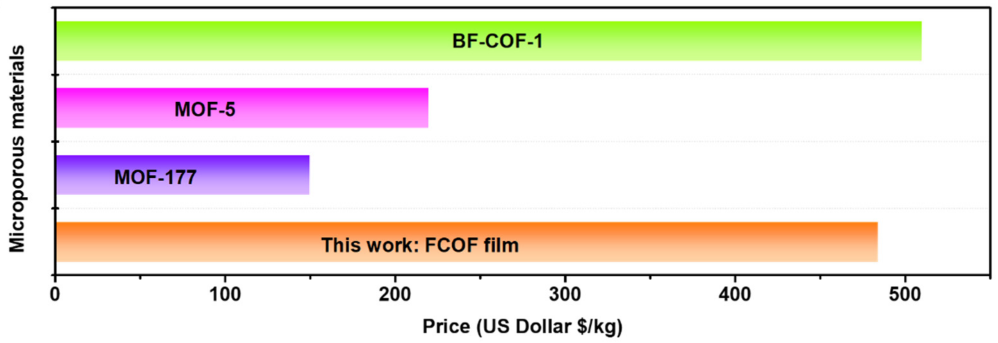

Supplementary Table 1: The overall cost estimation for the FCOF film. a. Detailed price calculation list (100 nm thickness FCOF film is chosen as an example). b. Production cost comparison of representative microporous materials (*Advanced Materials* (2021): 2102415. DOI: 10.1002/adma.202102415).

Note: The estimate for cost for FCOF film is based on laboratory-grade synthesis methods. The synthesis of COFs is closely related to the specific process(es), steps, and equipment used, so there are likely steps for actual optimization. For example, for small-scale trial production the purchasing prices of raw chemicals are relatively high per gram. When scaling the experiment to industry-scale, the purchasing prices of the raw chemicals per kilogram are reduced significantly *i.e.* the prices for TAPB, TFTA,

Dio, and Mes are reduced from, respectively, \$61.7 /g to \$9.0 /g; \$77.0 /g to \$14.6 /g; \$28.8 /100 ml to \$2.1 /100 ml, and; \$30.5 /100 ml to \$4.1 /100 ml. Therefore, production cost of FCOF can be significantly reduced from \$483 /kg to \$78 /kg. By synthesizing on a large-scale therefore the total-cost of COFs is reduced for practical commercialization

(a)

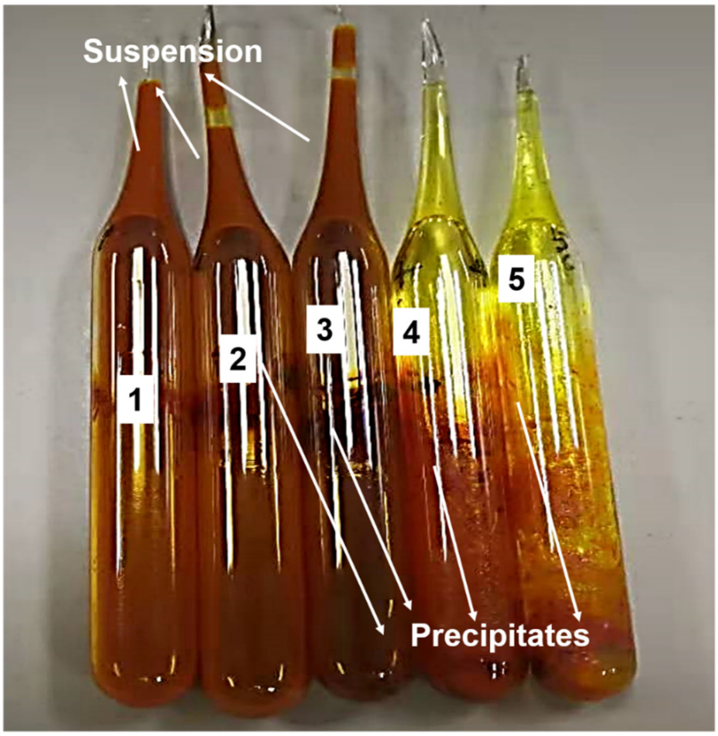

(b)

Trials	Solvent condition	Catalyst	Results	Suspension in the tube
1	Dio (1.8 mL), TMB (0.2 mL)	1.5 M HOAc (0.1 mL)	continuous film without precipitates	yes
2	Dio (1.8 mL), TMB (0.2 mL)	3 M HOAc (0.1 mL)	continuous film with few precipitates	yes
3	Dio (1.8 mL), TMB (0.2 mL)	6 M HOAc (0.1 mL)	discontinuous film with a large amount of precipitates	yes
4	Dio (1 mL), TMB (1 mL)	1.5 M HOAc (0.1 mL)	precipitates	no
5	Dio (0.2 mL), TMB (1.8 mL)	1.5 M HOAc (0.1 mL)	precipitates	no

Supplementary Fig. 1: Comparison of the synthesis conditions of the FCOF films. **a:** Photos of the solvothermal glass tubes with different reaction conditions and **b:** Corresponding details of the ingredients and obtained products.

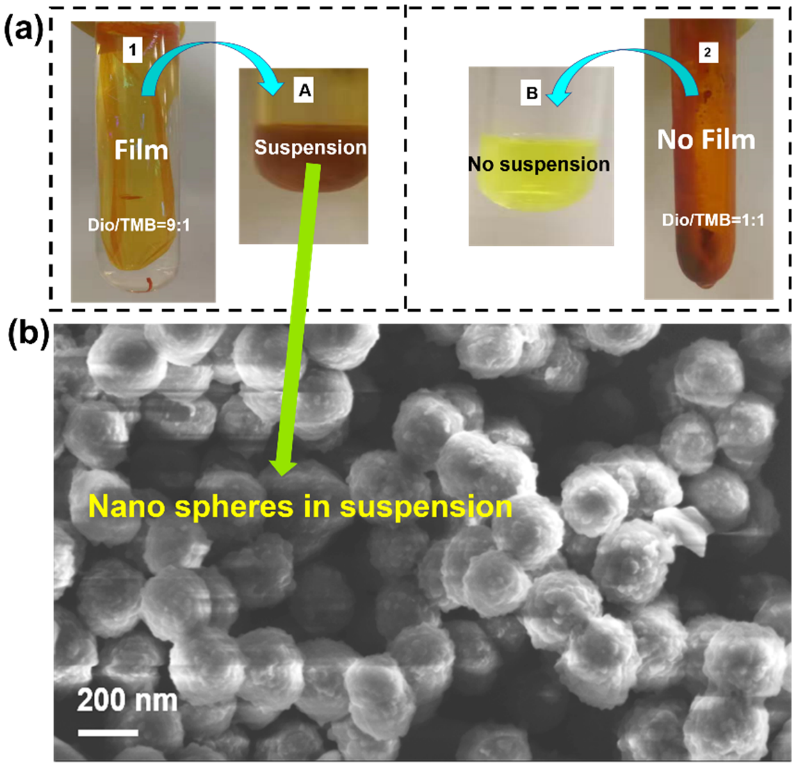

Supplementary Fig. 2: Product from different syntheses. a. Photograph of solvothermal glass tubes showing products (1 and 2) obtained from two reaction conditions (solvent ratio of Dio and TMB of A and B is 9:1 and 1:1, respectively, other conditions remain the same) and solutions (A and B) taken from the tubes following reaction. **b.** FESEM image showing uniform-sized nanospheres in suspension obtained from A solution. The finding confirms that solvent ratio of Dio and TMB is important to obtain nanospheres in suspension, and a crucial parameter in forming FCOF film.

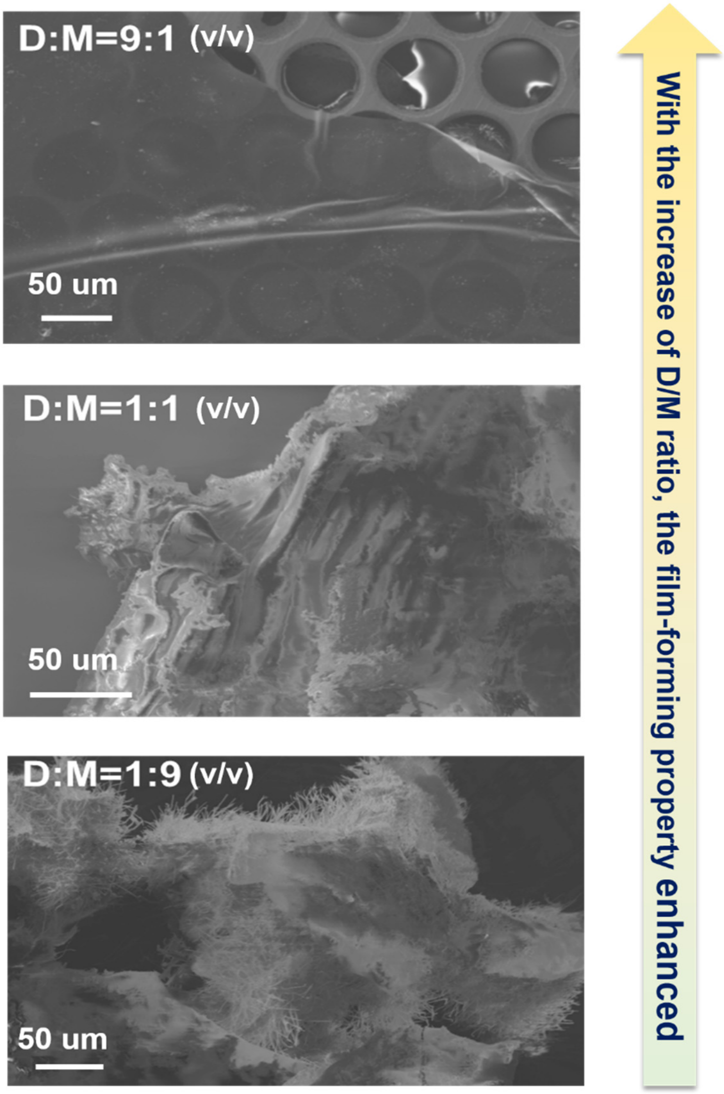

Supplementary Fig. 3: FESEM images of the FCOF films prepared with different D/M ratios. The results show that increasing the D/M ratio is important to achieve a high-quality film.

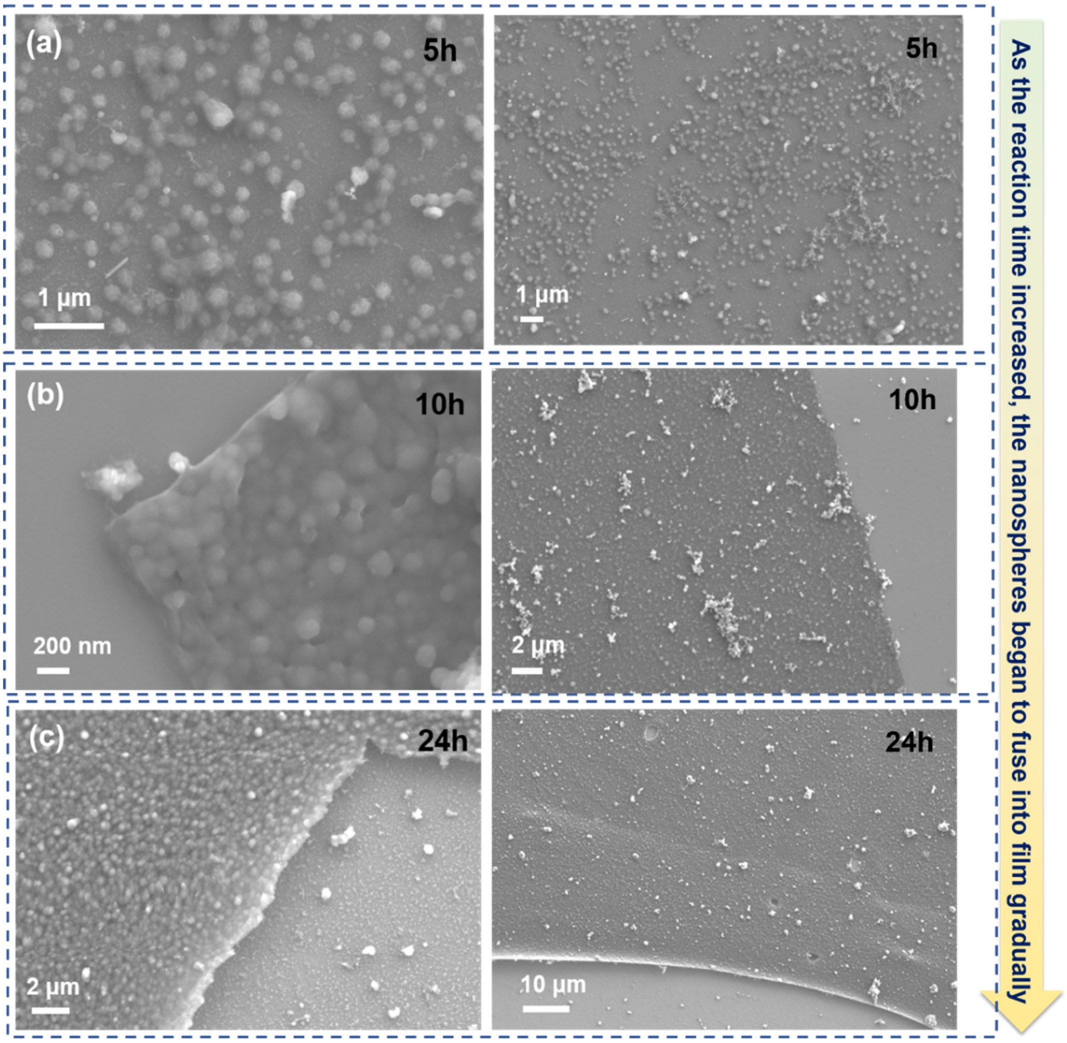

Supplementary Fig. 4: FESEM images of FCOF film grown on silicon substrate following reaction time. Results evidence the growth mechanism of FCOF film is based on assembly and fusion of nanospheres suspended in reaction solution.

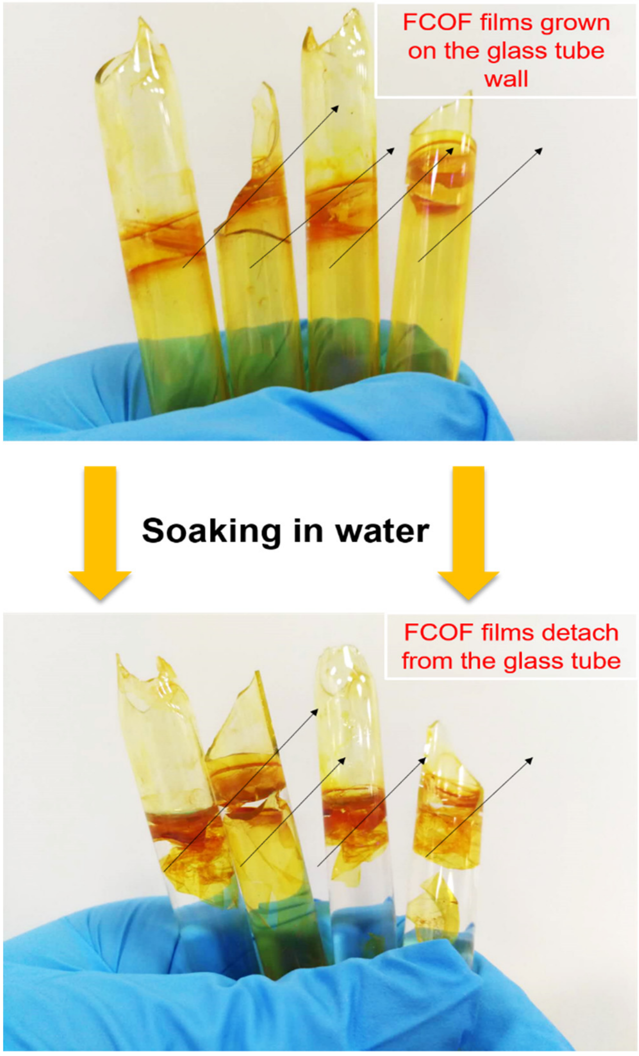

Supplementary Fig. 5: The transferring process of the FCOF film from the glass tubes.

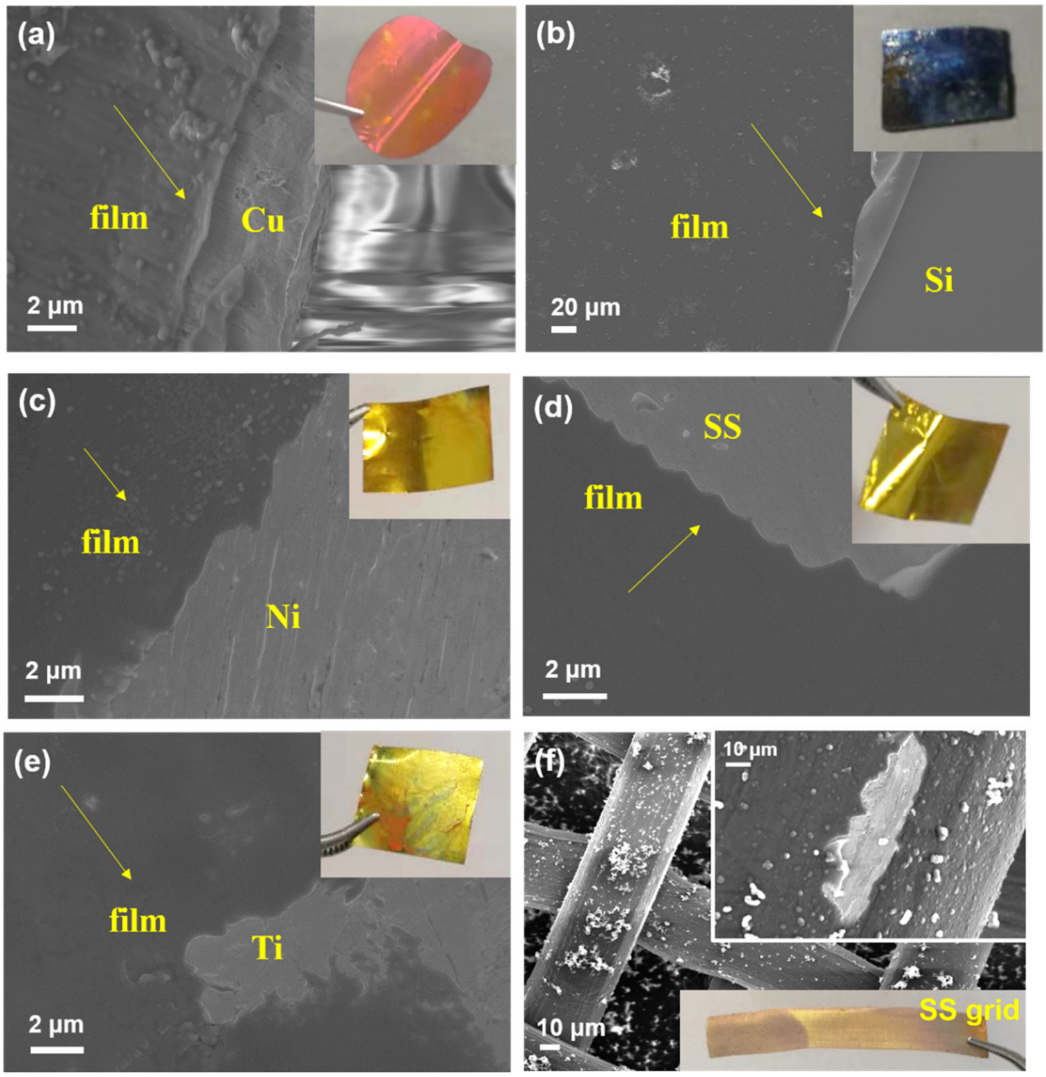

Supplementary Fig. 6: Photos and FESEM images of the FCOF films grown on various substrates including Cu, Si, Ni, Stainless Steel (SS), Ti and SS grid.

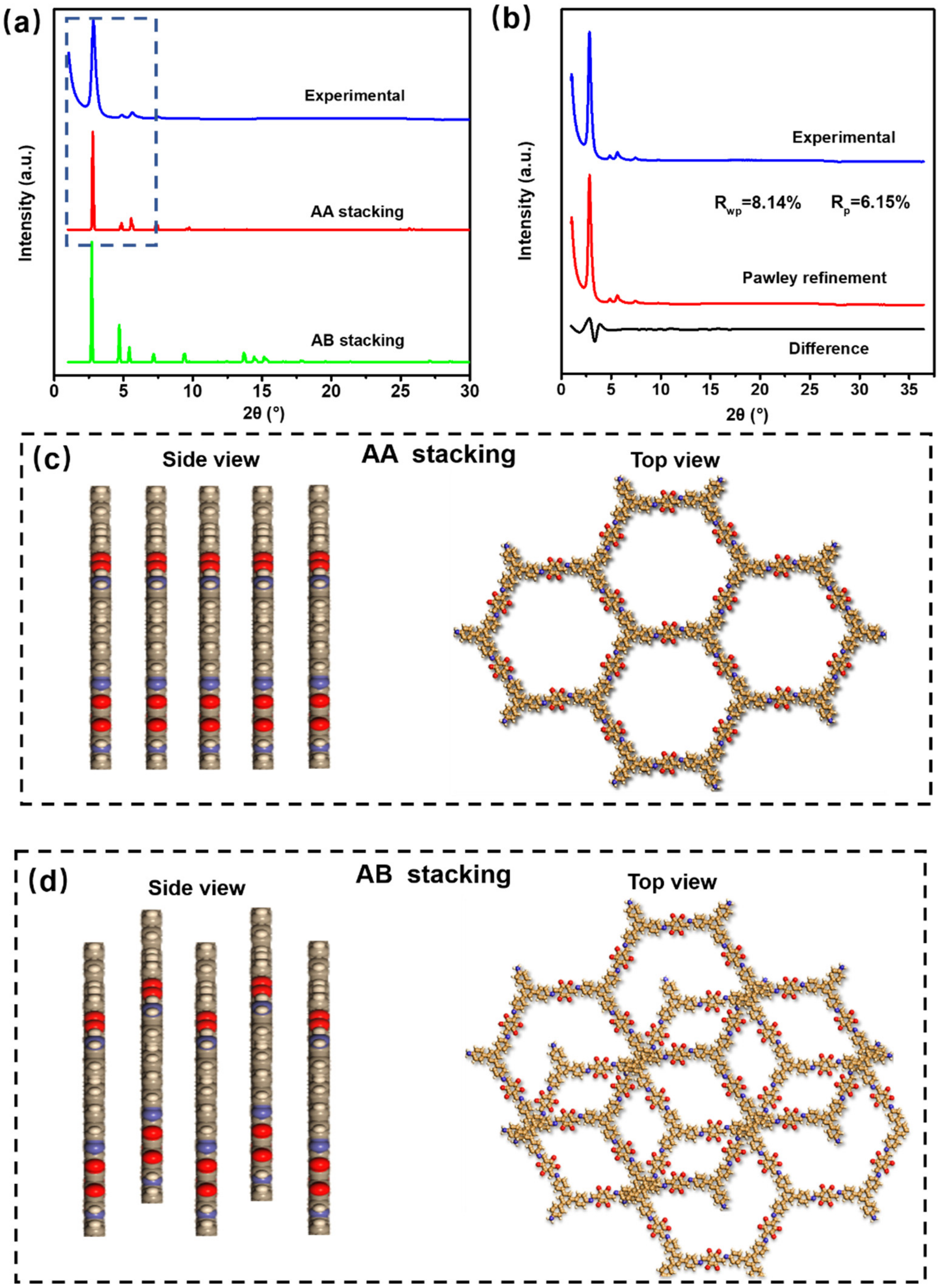

Supplementary Fig. 7: Stacking mode analysis for FCOF based XRD patterns. a. XRD patterns for FCOF showing experiment and simulation based on AA and AB stacking mode. **b.** Comparison of XRD patterns of experiment and Pawley-refined results. Top and side view of the unit cell of FCOF showing **c.** AA and d. AB stacking mode.

Analysis following Supplementary Fig. 7: To determine the crystal structure of π-π stacking mode in FCOF, theoretical simulation and Pawley refinement are carried out (Supplementary Fig. 7). According to the hcp topology, the possible structure of FCOF is constructed by using two typical stacking modes i.e. AA and AB stacking (Supplementary Figs. 7c and d). The XRD patterns for the two stacking modes are obtained by simulation (Supplementary Fig. 7a). The findings confirm that the degree of matching of XRD for AA stacking mode and experiment, is better than for AB stacking. Therefore, AB stacking mode is excluded. Additionally, Pawley refinement is used to optimize the most likely structure for FCOF (AA stacking). (Supplementary Fig. 7b). The difference between the obtained and predicted unit cell parameters is negligible with R_{wp}: 8.14 %, R_p: 6.15 %, evidencing AA stacking in FCOF.

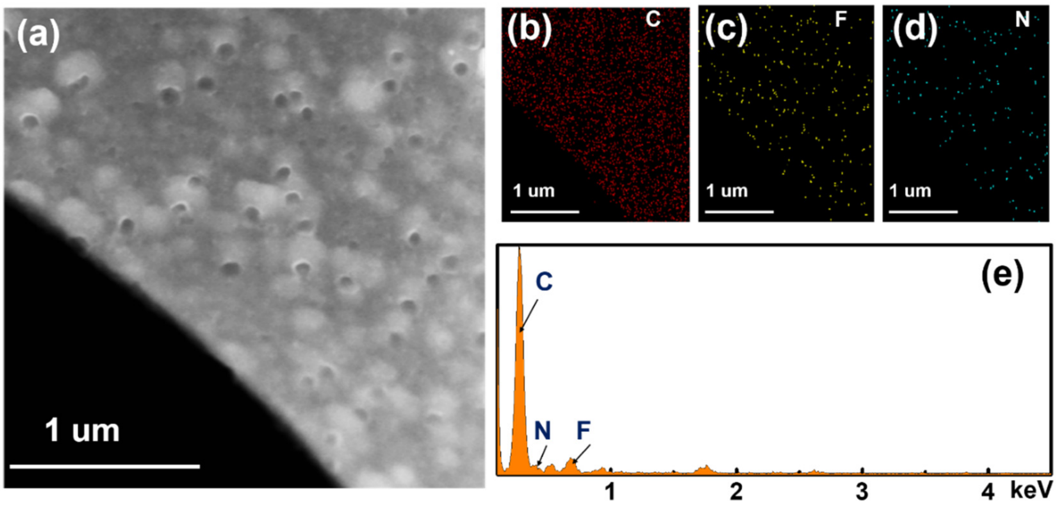

Supplementary Fig. 8: TEM image of the FCOF film and the corresponding EDX results of C, F, and N elements.

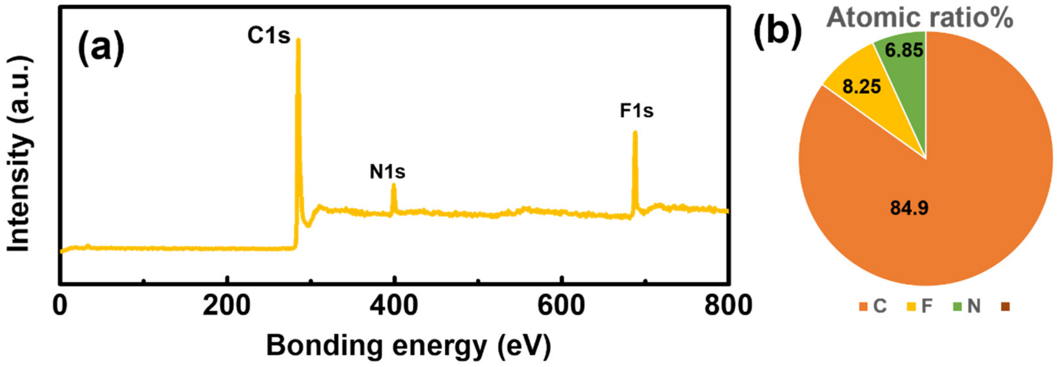

Supplementary Fig. 9: XPS full energy spectrum of FCOF film and atomic ratio of C, N, and F elements.

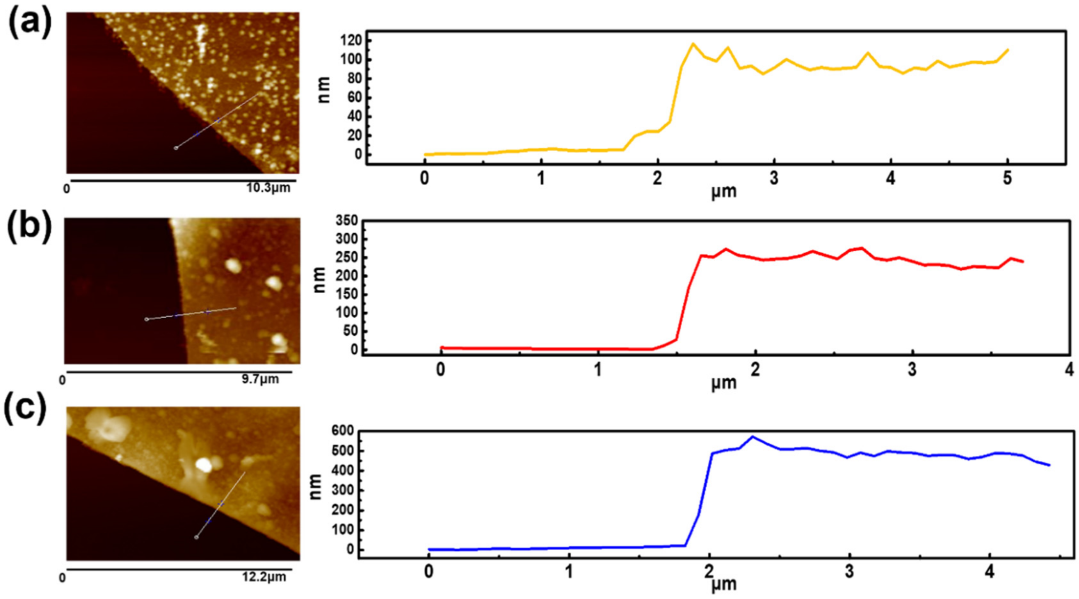

Supplementary Fig.10: AFM results of the FCOF films. Three different thickness: **a.** ~100nm, **b.** ~250nm and **c.** ~500nm of the films are prepared**.**

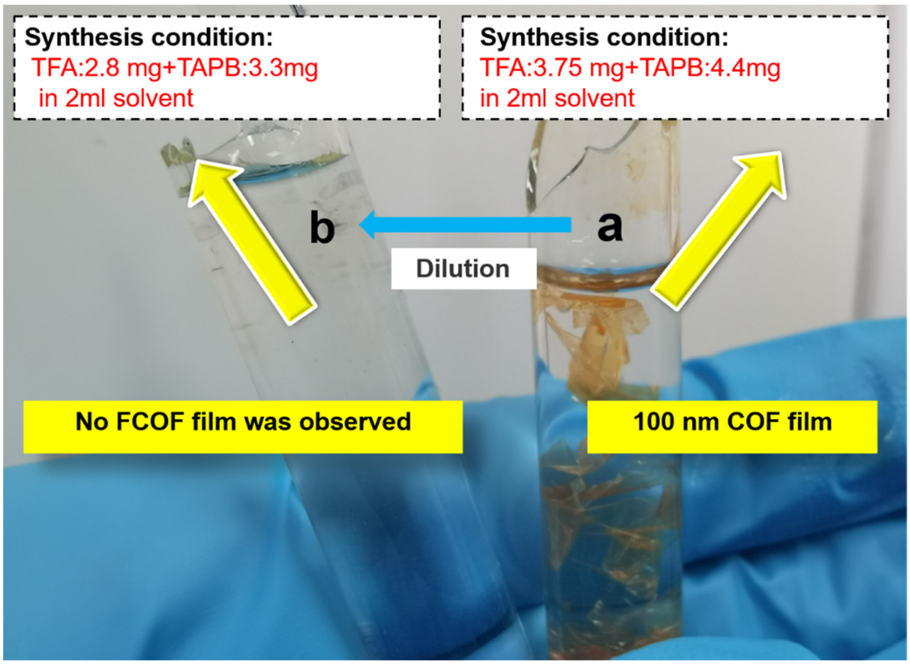

Supplementary Fig. 11: Photographs showing concentration of monomers for preparing ~ a. 100-nm-thick film**; b.** when diluted, no film observed.

Analysis: In the solvothermal process the growth mechanism of the film is mainly attributable to the fusion of many nanospheres formed in the condensation reaction (Supplementary Figs. 2b and 4). The thickness of the film is therefore controlled by the concentration of nanospheres in the reaction solvent. When the concentration of nanospheres reaches its lower threshold, the amount of nanospheres formed in the solvent is therefore not sufficient to fuse on the hydrothermal tube-wall to form a continuous film. For example, as is shown in Supplementary Fig. 4a, in the early stage of the reaction (5 h), the number of nanospheres is low and not able to form a continuous film. A limit to minimum thickness therefore exists for the FCOF film.

As a result, a limit to minimum thickness exists for preparing FCOF film. When the concentration of monomers is diluted, no film occurs. Therefore, in this experiment, to

suppress the side reactions and retard Zn dendrites, a reliable film with a thickness of ~ 100 nm is prepared.

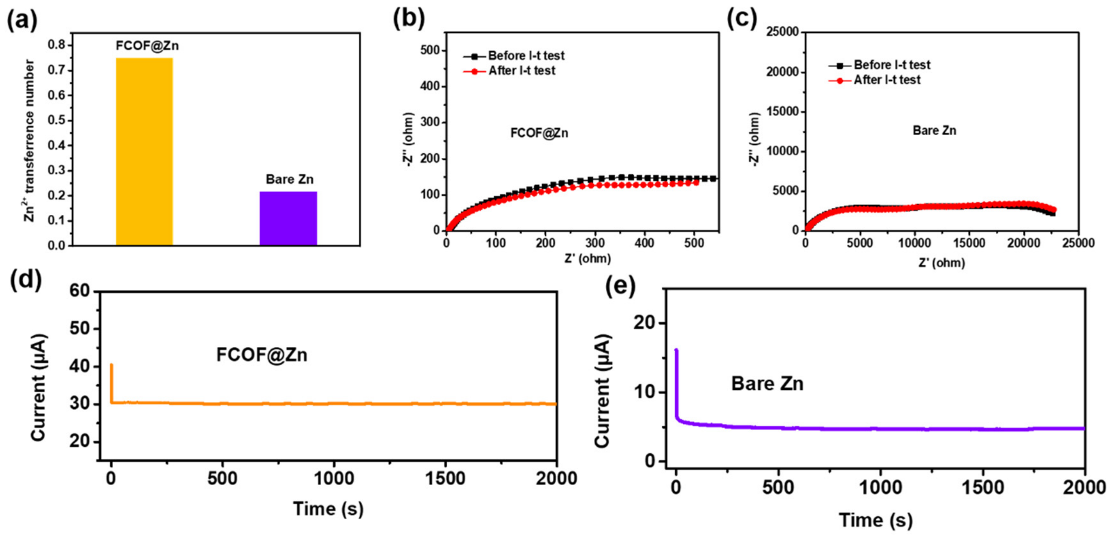

Supplementary Fig. 12: Computation of Zn^{2+} transference number (ZTN) based on chronoamperometric tests (*I-t*) under a polarization of 20 mV. a. Computed Zn^{2+} transference number (ZTN) for FCOF@Zn and bare Zn symmetric cells. EIS results for **b.** FCOF@Zn and **c.** bare Zn symmetric cells before and following polarization (*I-t* test). Note: The assembled batteries are rested for 24 h to ensure the impedance does not change, and the chronoamperometric (*I-t*) tests are conduct. Therefore, the impedance is greater than that for Supplementary Fig. 16c. *I-t* curves for **d.** FCOF@Zn and **e.** bare Zn symmetric cells. Because EIS findings remain nearly unchanged before and following polarization, ZTN can be computed from I/I_0, where I_0 represents initial current and I, equilibrium current.

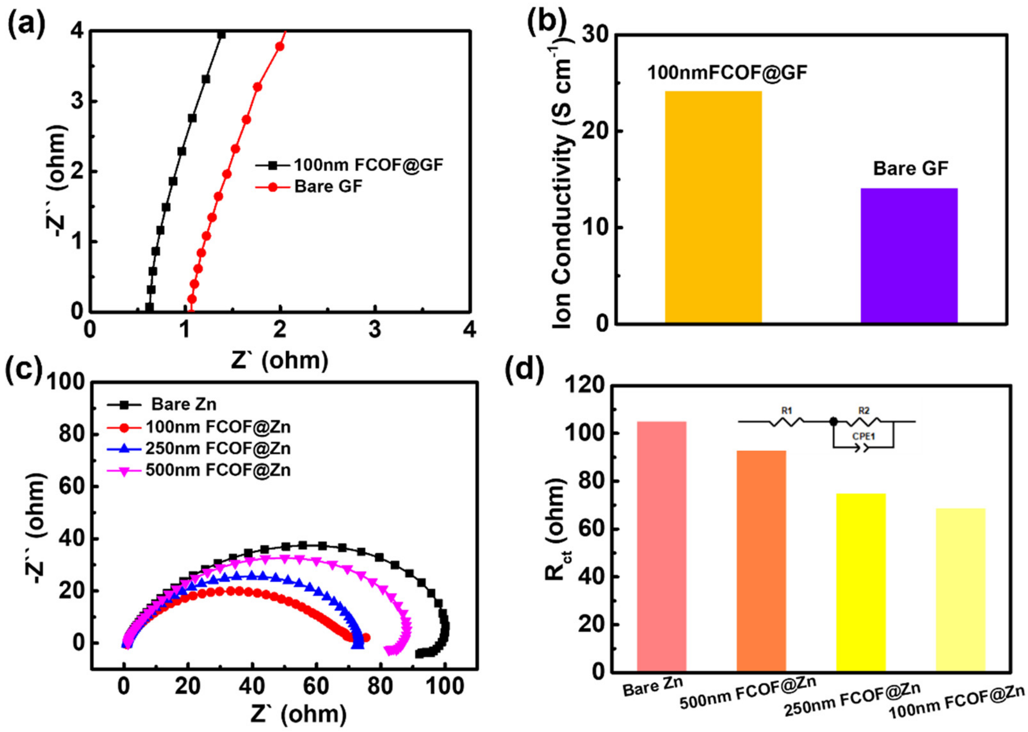

Supplementary Fig. 13: Characterization of Zn^{2+} ion transport behavior of the FCOF films. a. EIS results of the symmetric cells in SS/FCOF@GF or bare GF /SS configurations (immersed in 2 M $ZnSO_4$). **b.** Calculated ion conductivity based on the EIS results. **c.** The EIS results of the symmetric cells in FCOF@Zn/FCOF@Zn configurations. **d.** Simulated R_{ct} of the symmetric cells according to the equivalent circuit (inset).

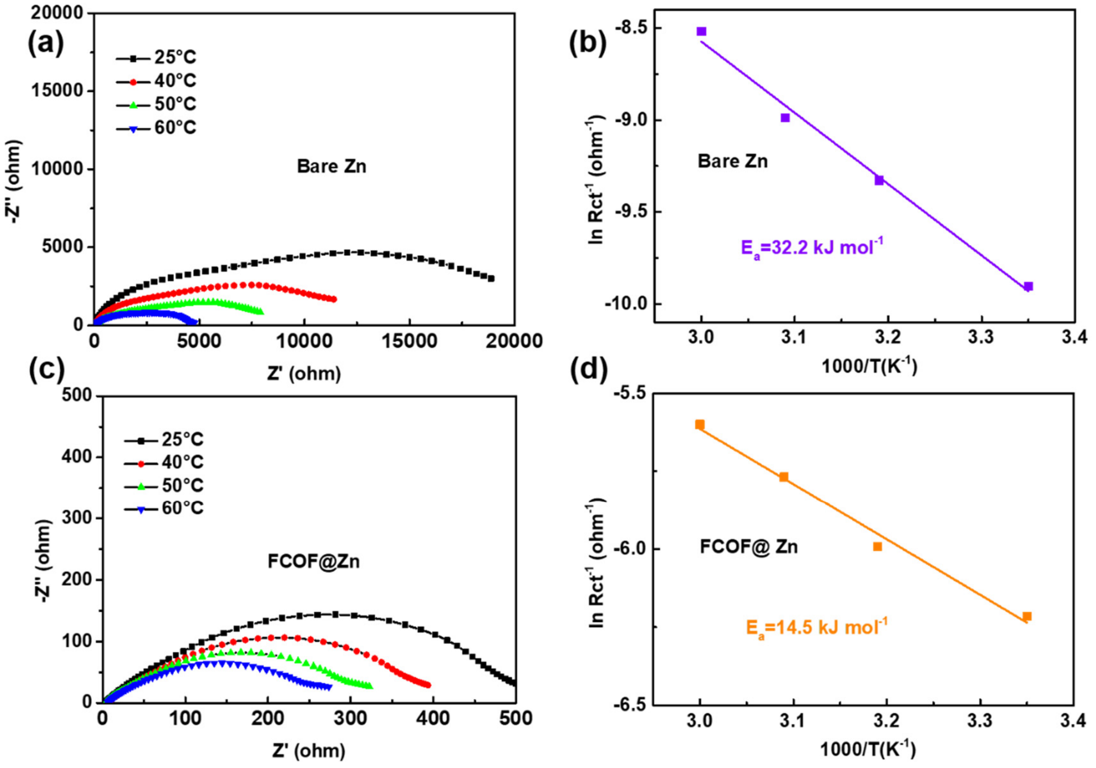

Supplementary Fig. 14: E_a based on temperature-dependent EIS. EIS plots for symmetrical cells of **a.** FCOF@Zn and **c.** Bare Zn under different temperatures. Corresponding Arrhenius curves and computed E_a for **b.** FCOF@Zn and **d.** Bare Zn.

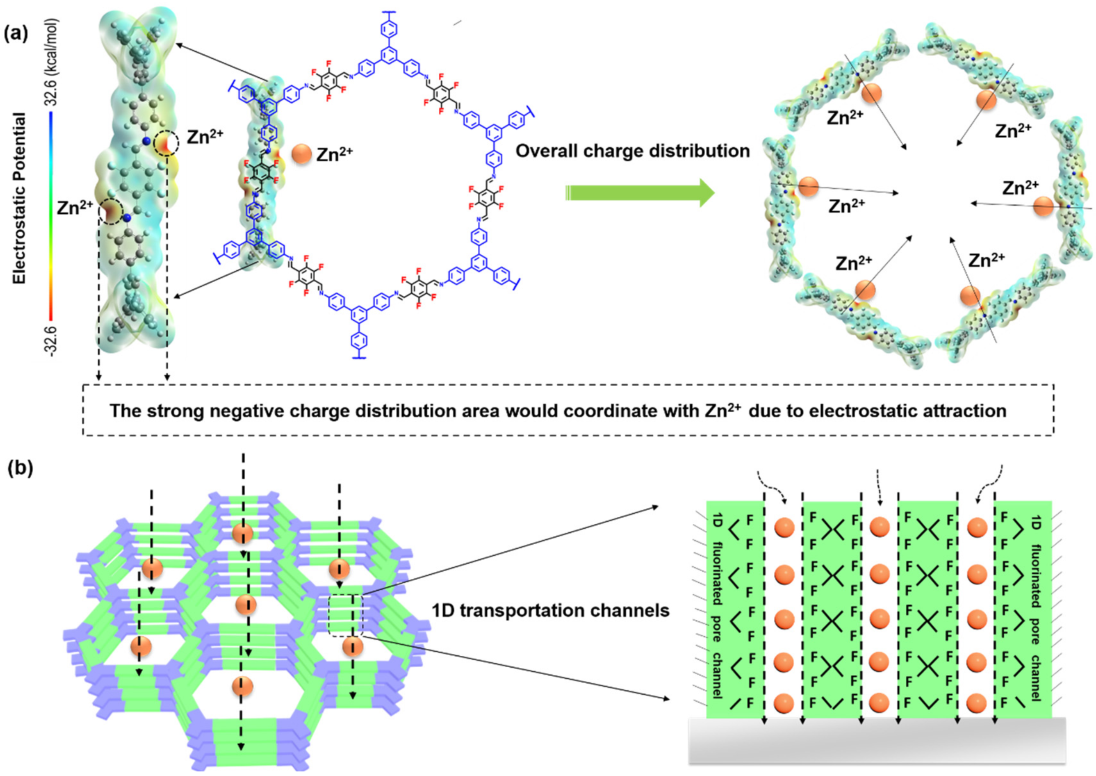

Supplementary Fig. 15: Mechanism for ion transport acceleration in FCOF. a. Charge density distribution computed from first-principles for repeated chemical unit in FCOF. **b.** Schematic showing positively charged Zn^{2+} facilely hopping around F atom sites and transport along 1D aligned channels to shorten ion transportation pathways.

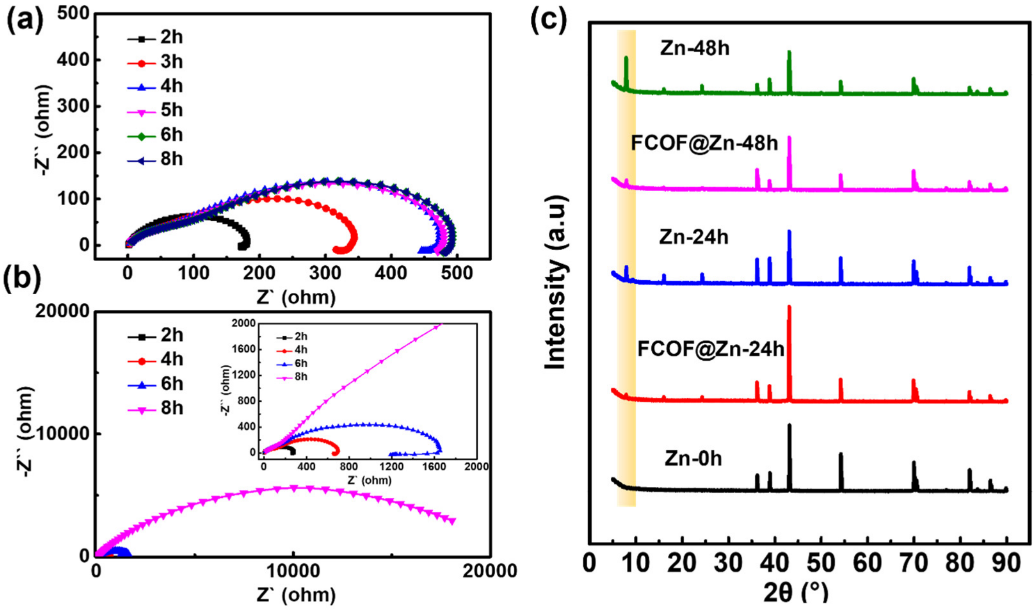

Supplementary Fig. 16: Comparison of the electrolyte anticorrosion property of the FCOF@Zn and bare Zn anodes. Time-dependent EIS results of the **a.** FCOF@Zn or **b.** bare Zn symmetric cells. **c.** Time-dependent XRD patterns of the FCOF@Zn and bare Zn anodes after immersing in 2M $ZnSO_4$.

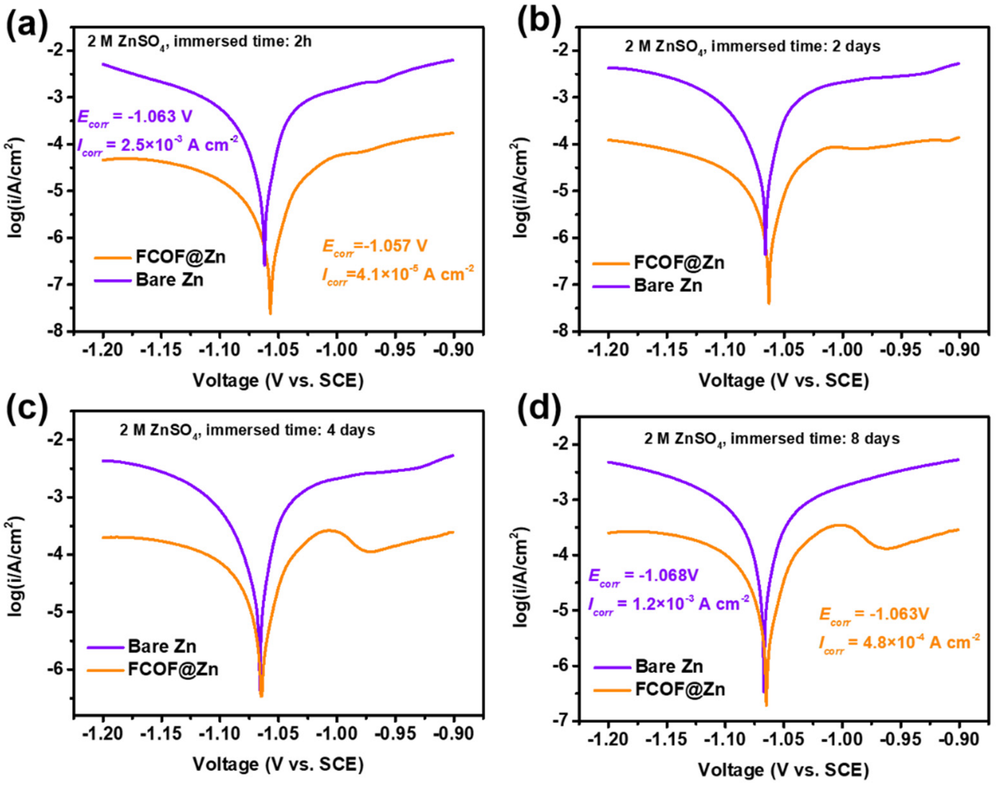

Supplementary Fig. 17: Time-dependent polarization curves for Zn and FCOF@Zn electrodes in 2 M $ZnSO_4$ electrolyte. Immersion times are **a.** 2 h, **b.** 2 days, **c.** 4 days and **d.** 8 days.

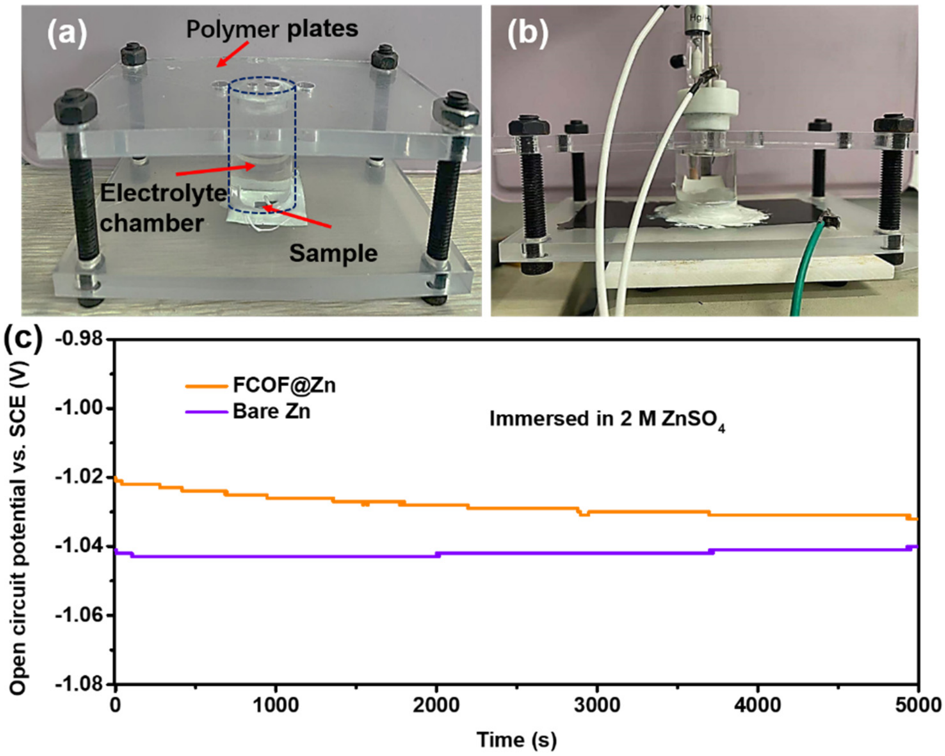

Supplementary Fig. 18: Corrosion test of Zn anodes. a. Home-made apparatus and **b.** Corrosion tests on the CHI660E electrochemical workstation. **c.** Open circuit potentials for bare Zn and FCOF@Zn electrodes in 2 M $ZnSO_4$ electrolyte.

Note: Home-made apparatus assembly steps are: waterproof thermosetting resin is used to enable the metal anode samples to fix in the center of the polymer plate. An additional polymer plate, with a circular electrolyte cell, is used to cover the upper surface of samples. The two plates are bolted together. Waterproof thermosetting resin is used to seal the edge between the samples and the circular electrolyte cell, to ensure no leakage occurs. The electrolyte is injected and three-electrode is inserted for testing.

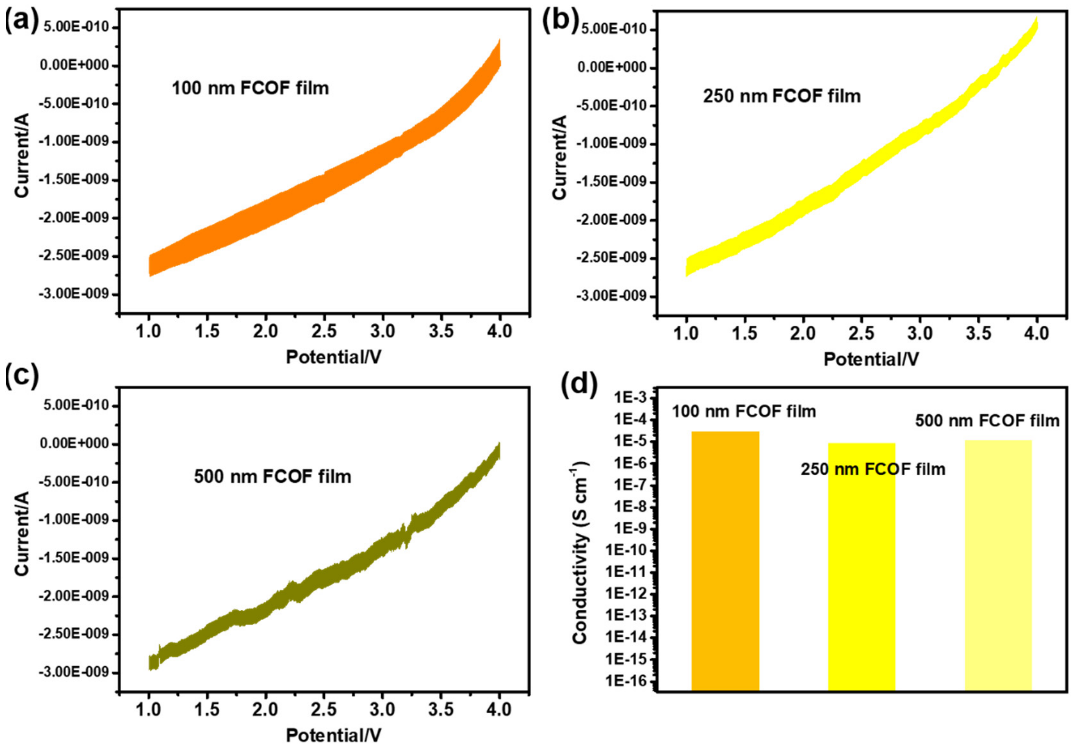

Supplementary Fig. 19: Electronic conductivity of FCOF films determined from *I-V* curves. *I-V* curves for **a.** 100 nm film, **b.** 250 nm film and **c.** 500 nm film. **d.** Corresponding computed electronic conductivity.

Results confirm that the *I-V* slopes of films with different thicknesses (100 nm, 250 nm, and 500 nm) exhibit similar characteristics, with an electronic conductivity of, respectively, 3×10^{-5}, 9×10^{-6} and 1.2×10^{-5} S cm^{-1}.

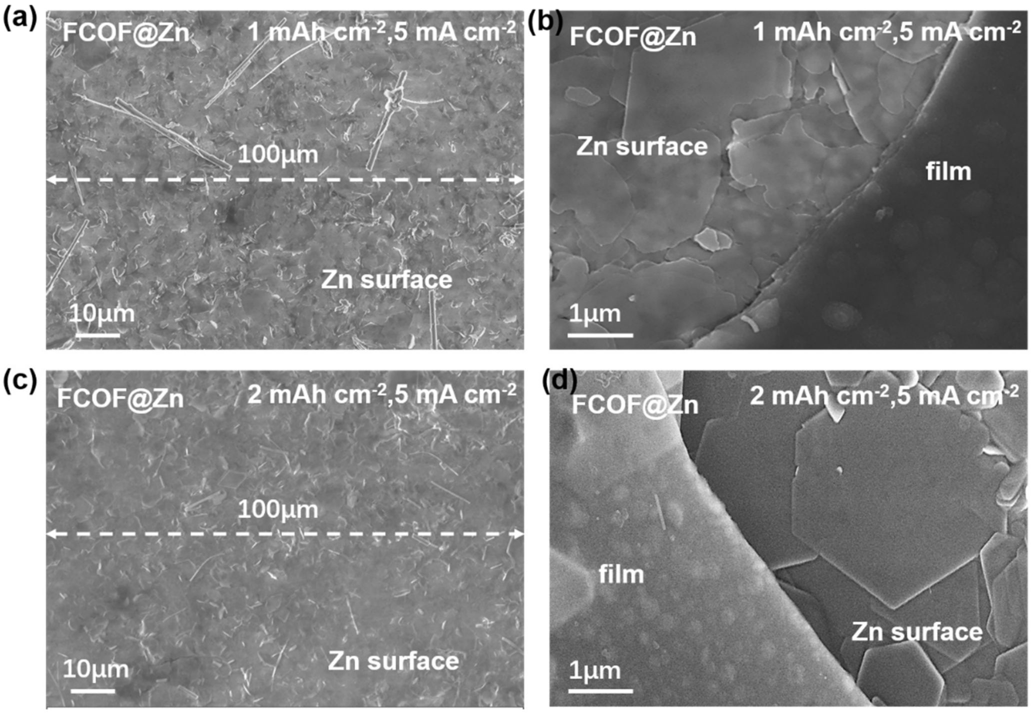

Supplementary Fig. 20: SEM images with low and high magnification revealing Zn deposition morphology on FCOF film coated Zn. a. and **b.** Deposition morphology under capacity of 1 mA h cm^{-2}. **c.** and **d.** Deposition morphology under capacity of 2 mA h cm^{-2}.

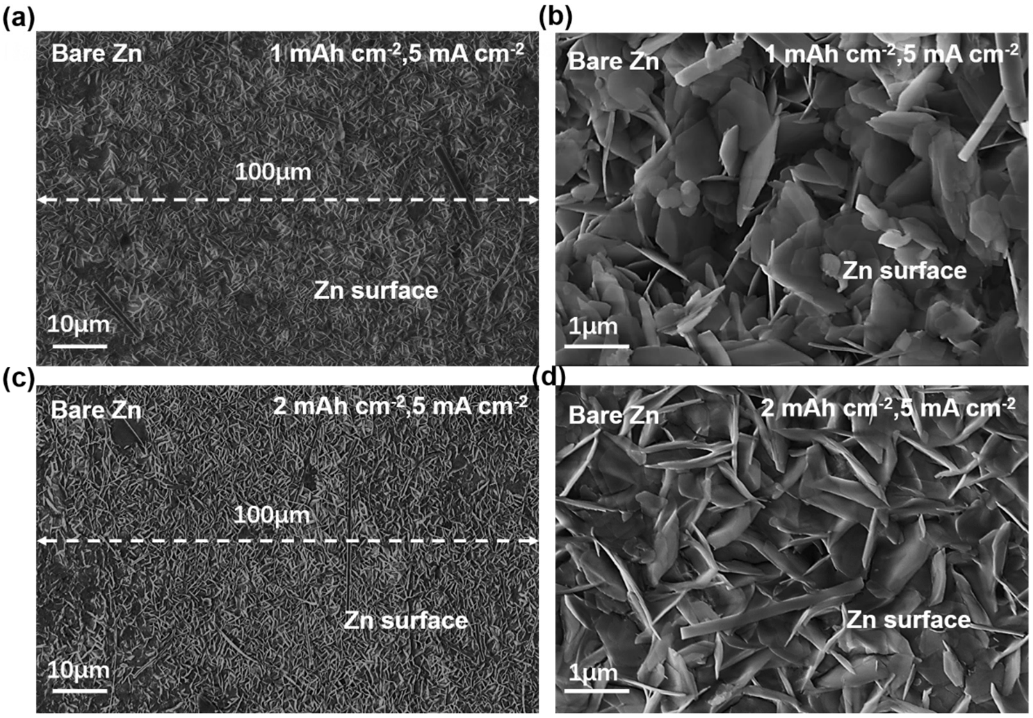

Supplementary Fig. 21 SEM images with low and high magnification revealing large-scale Zn deposition morphology on bare Zn. a. and **b.** Deposition morphology under capacity of 1 mA h cm^{-2}. **c.** and **d.** Deposition morphology under capacity of 2 mA h cm^{-2}.

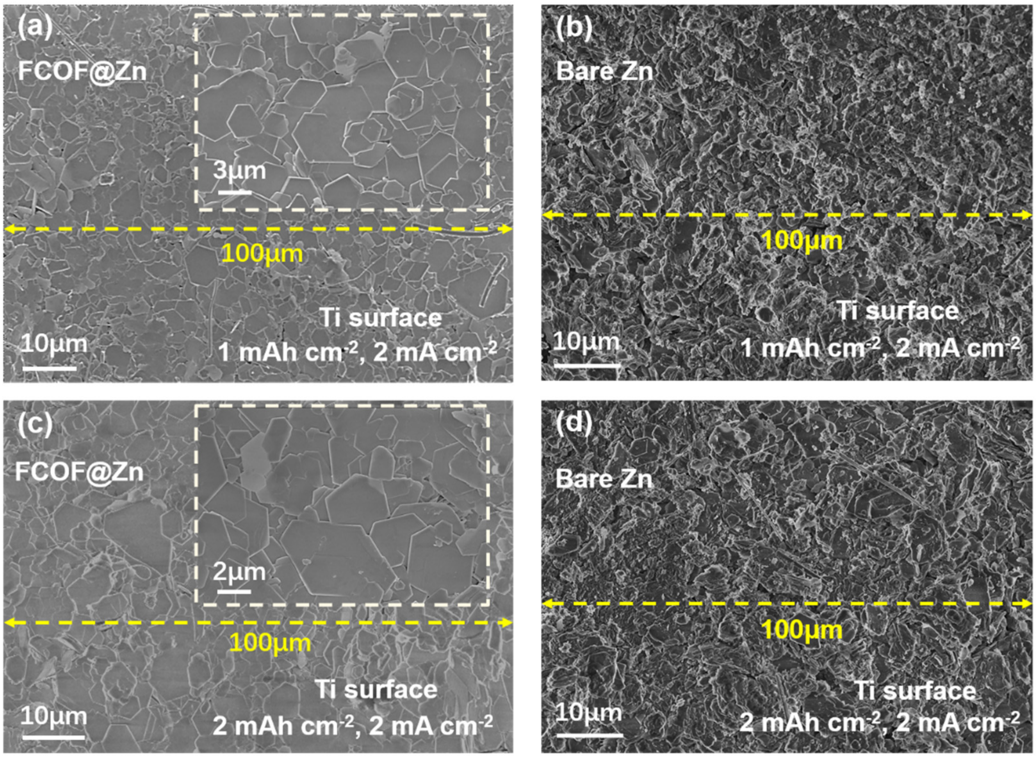

Supplementary Fig. 22: SEM images with low magnification revealing large-scale Zn deposition morphology on Ti-foil. a. and **c.** Deposition morphology underneath FCOF film. **b.** and **d.** Deposition morphology on bare Ti-foil. (Insets in **a.** and **c.** show high-magnification images of horizontally arranged Zn platelets).

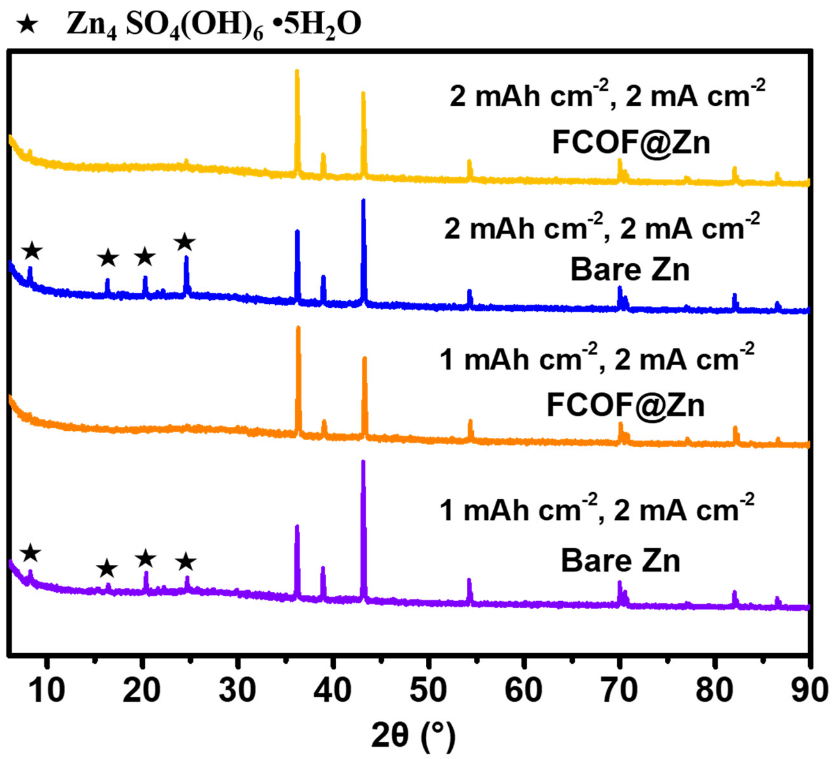

Supplementary Fig. 23: XRD patterns for initial FCOF@Zn deposits and bare Zn deposits. Deposition capacities of 1 mAh cm^{-2} and 2 mAh cm^{-2}, respectively.

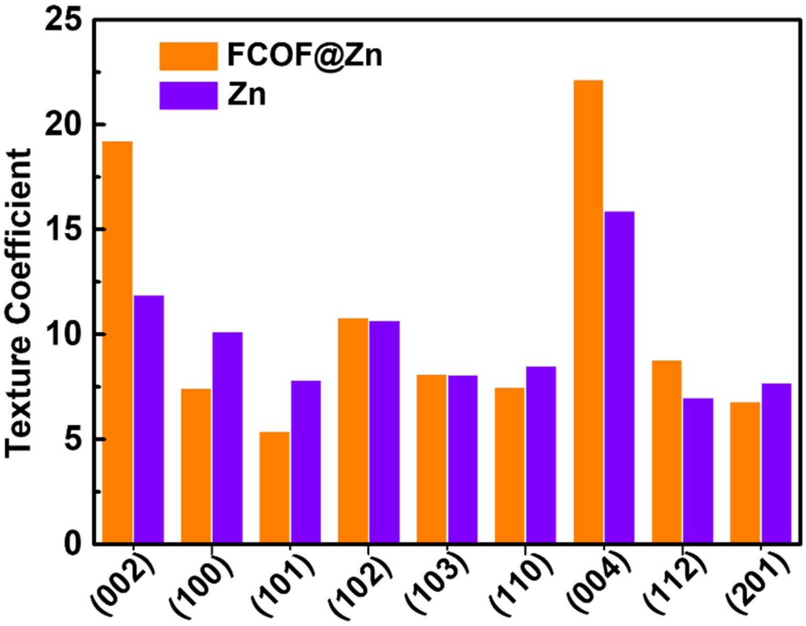

Supplementary Fig. 24: The calculated texture coefficients of different crystal planes of Zn.

The preferred orientations of Zn deposits can be determined calculating the texture coefficient (T_c) according to equation (*J. Mater. Sci. Technol.* 31, 175-181 (2015)):

$$T_c = \frac{I_{(hkl)}/I_{0(hkl)}}{\frac{1}{n}\sum(I_{(hkl)}/I_{0(hkl)})}$$

where $I_{(hkl)}$ is the peak intensity of zinc electrodeposits and $\sum I$ is the sum of the intensities of independent peaks. The index 0 refers to the intensities for the standard Zn sample taken from a powder diffraction file (PDF) card (PDF# 99-0110). n is the number of diffraction planes considered in the analysis.

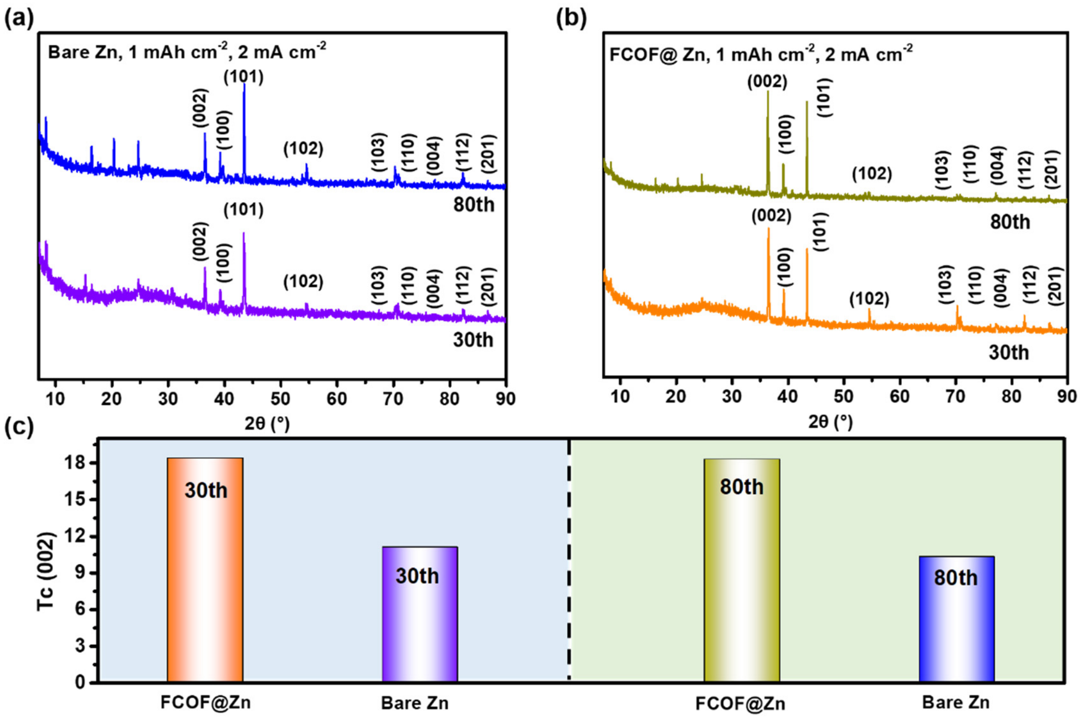

Supplementary Fig. 25: XRD patterns after cycling. a. Bare Zn deposits. **b.** Zn deposits underneath FCOF film following 30th and 80th cycles at 1 mAh cm^{-2} and 2 mA cm^{-2}. **c.** Computed texture coefficients for (002) crystal planes.

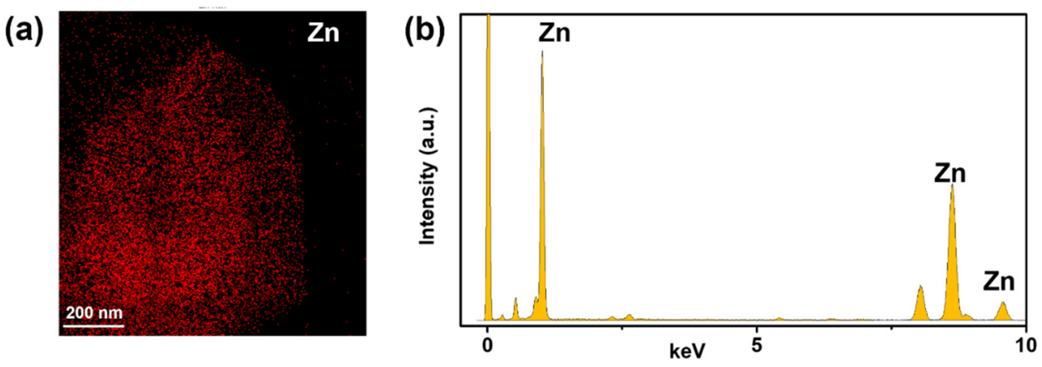

Supplementary Fig. 26: The EDX results of a Zn platelet after stripping off the FCOF film under HRTEM test.

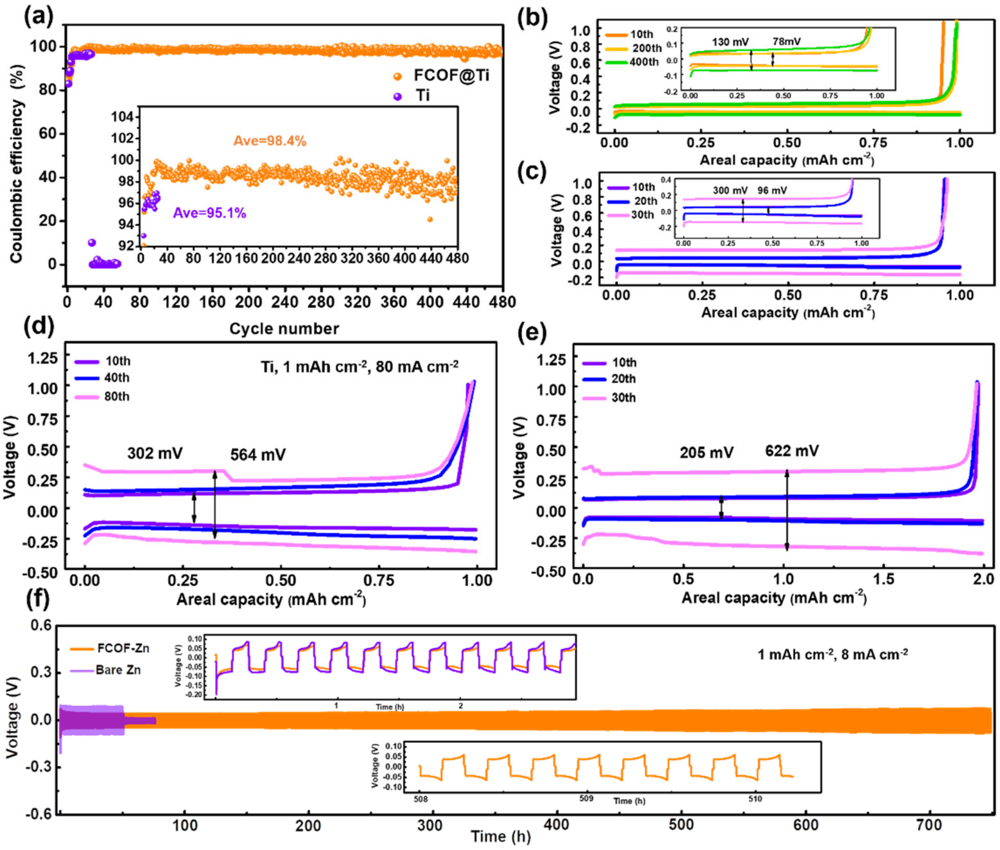

Supplementary Fig. 27: The electrochemical performance of Zn anodes. a. CE of Zn plating/stripping on FCOF film- coated Ti and bare Ti at 1 mAh cm^{-2}, 5 mA cm^{-2}. **b-e.** The voltage profiles at selected cycles. The insets are enlarged voltage profiles. **f**. Cycling performance of Zn symmetric cells with or without FCOF film protection at g: 1 mAh cm^{-2} and 8 mA cm^{-2}.

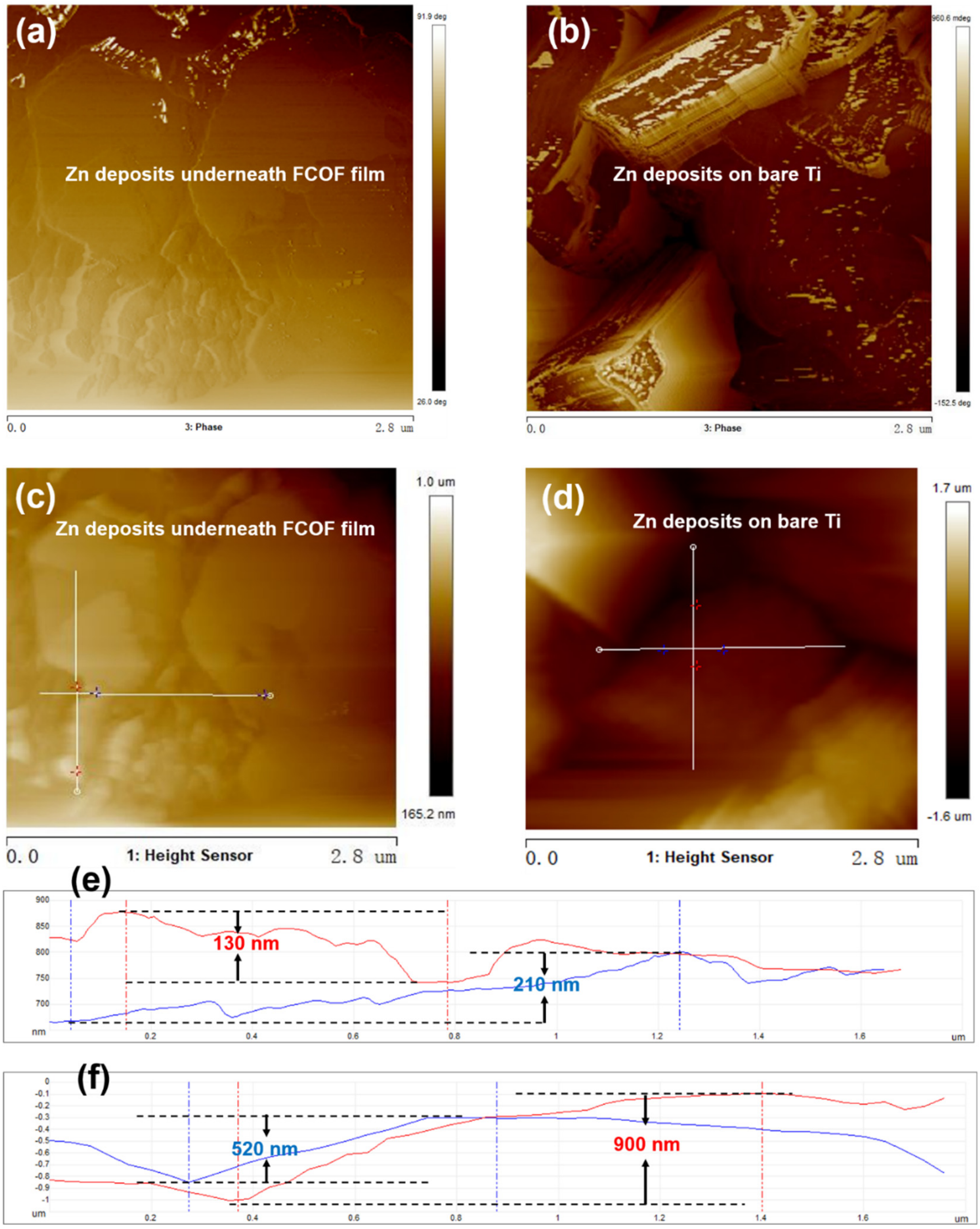

Supplementary Fig. 28: AFM characterization of Zn deposits after cycling (100 cycles at 1 mAh cm^{-2}, 5 mA cm^{-2}). a-b. Phase and **c-d.** height imaging of Zn deposits on FCOF film coated or bare Ti. Height profiles along X (blue) and Y(red) axis of Zn deposits on **e**. FCOF film coated or **f**. bare Ti.

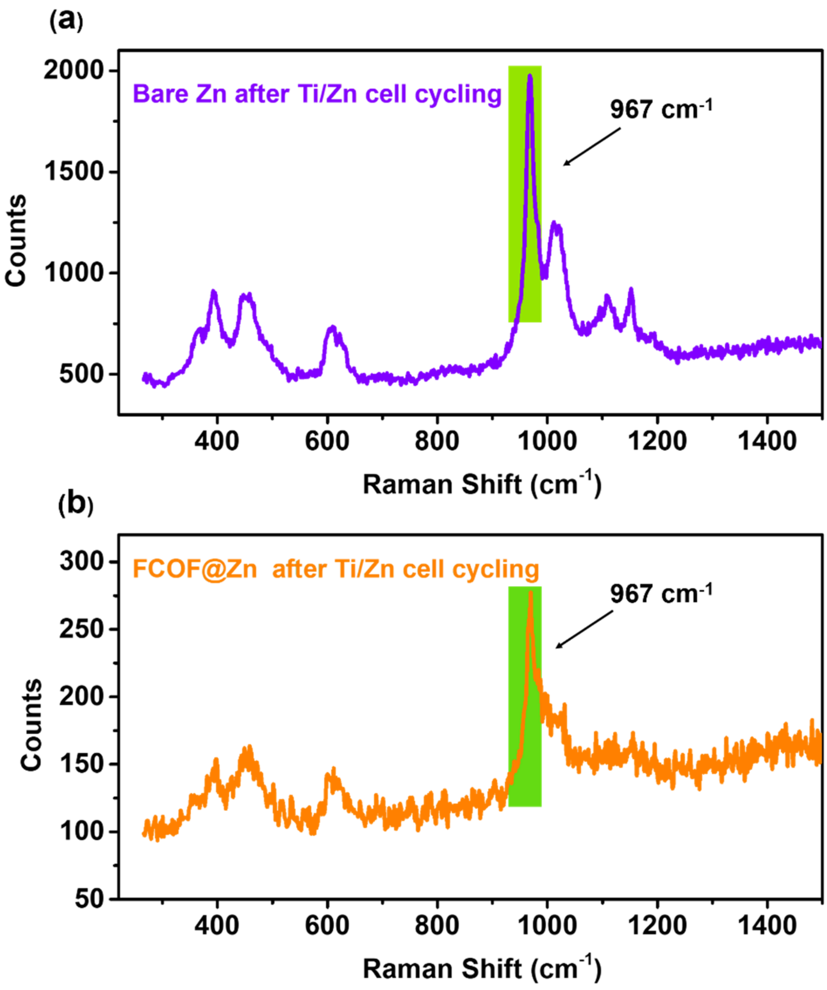

Supplementary Fig. 29: Raman spectra of the Zn deposits after cycling on a. bare Ti and **b**. FCOF coated Ti. The dominant peak at 967 cm^{-1} is chosen to show the Raman mapping.

Ref (Ti/Zn asymmetric cell)	*Capacity*	*Current Density*	*Cycle number*
Angew. Chem. Int. Ed. 2021, 60, 2–9	1 mAh cm^{-2}	0.5 mA cm^{-2}	50
Angew.Chem. Int. Ed. 2020, 59,19292 –19296	0.5 mAh cm^{-2}	1 mA cm^{-2}	300
J. Mater. Chem. A, 2021, 9, 5597-5605	0.5 mAh cm^{-2}	/	300
Nano-Micro Lett. (2021) 13:79	5 mAh/cm^2	5 mA cm^{-2}	14
Energy Environ. Sci., 2019, 12, 1938-1949	0.4 mAh cm^{-2}	0.4 mA cm^{-2}	300
Advanced Functional Materials, 2020, 30(13): 1908528.	1 mAh cm^{-2}	5 mA cm^{-2}	210
Materials Today Energy 18 (2020) 100563	2.5 mAh cm^{-2}	2.5 mA cm^{-2}	60
Advanced Functional Materials, 2021, 31(2): 2001867.	0.885 mAh cm^{-2}	1.77 mA cm^{-2}	1000
J. Am. Chem. Soc. 2020, 142, 21404−21409	0.5 mAh cm^{-2}	1 mA cm^{-2}	400
Energy Storage Materials 27 (2020) 1–8	0.5 mAh cm^{-2}	0.5 mA cm^{-2}	50
Advanced Materials, 2020, 32(33): 2003425.	1 mAh cm^{-2}	2 mA cm^{-2}	20
Journal of Materials Chemistry A, 2020, 8(34): 17725-17731.	/	1 mA cm^{-2}	300
Nature communications, 2019, 10(1): 1-12.	1 mAh cm^{-2}	0.5 mA cm^{-2}	45
**Present work**	**1 mAh cm^{-2}**	**5 mA cm^{-2}**	**480**
**Present work**	**1 mAh cm^{-2}**	**80 mA cm^{-2}**	**320**
**Present work**	**2 mAh cm^{-2}**	**40 mA cm^{-2}**	**260**

Supplementary Table 2: Cycle number and current density comparison of CE of Zn plating/stripping for the reported Zn aqueous batteries.

Ref (symmetric cell)	*Capacity*	*Current Density*	*Cycle life*
ACS Energy Letters, 2020, 5(9): 3012-3020.	2 mAh cm^{-2}	10 mA cm^{-2}	400 h
ACS Energy Lett. 2021, 6, 395−403	0.067 mAh cm^{-2}	0.2 mA cm^{-2}	170 h
ACS Energy Lett. 2021, 6, 675−683	0.1 mAh cm^{-2}	5 mA cm^{-2}	370 h
Adv. Energy Mater. 2021, 11, 2003419	1 mAh cm^{-2}	4 mA cm^{-2}	116 h
Adv. Funct. Mater. 2021, 31, 2006495	0.1 mAh cm^{-2}	0.1 mA cm^{-2}	200 h
Adv. Mater. 2021, 33, 2007497	1 mAh cm^{-2}	4 mA cm^{-2}	300 h
Adv. Mater. 2021, 2007416	1 mAh cm^{-2}	5 mA cm^{-2}	220 h
Adv. Mater. 2021, 2007388	1 mAh cm^{-2}	1 mA cm^{-2}	800 h
Adv. Mater. 2021, 2007406	1 mAh cm^{-2}	5 mA cm^{-2}	2500 h
Energy Storage Materials 36 (2021) 132–138	1 mAh cm^{-2}	2 mA cm^{-2}	400 h
Journal of Energy Chemistry 55 (2021) 549–556	0.5 mAh cm^{-2}	0.5 mA cm^{-2}	240 h
Nano-Micro Lett. (2021) 13:79	1 mAh cm^{-2}	1 mA cm^{-2}	2000 h
Nano Energy 80 (2021) 105478	0.5 mAh cm^{-2}	5 mA cm^{-2}	800 h
Angew. Chem. Int. Ed. 2021, 60, 2–9	0.2 mAh cm^{-2}	1 mA cm^{-2}	800 h
Nature communications, 2020, 11(1): 1-9.	1 mAh cm^{-2}	0.5 mA cm^{-2}	2000 h
Nature communications, 2020, 11(1): 1-7.	1 mAh/cm^2	1 mA cm^{-2}	460 h
Energy & Environmental Science, 2020, 13(2): 503-510.	1.25 mAh cm^{-2}	5 mA cm^{-2}	500 h
Nature materials, 2018, 17(6): 543-549.	0.033 mAh cm^{-2}	0.2 mA cm^{-2}	170 h
**Present work**	**1 mAh cm^{-2}**	**5 mA cm^{-2}**	**1700 h**
**Present work**	**1 mAh cm^{-2}**	**40 mA cm^{-2}**	**700 h**

Supplementary Table 3: Cycle life comparison of symmetric Zn cells in reported Zn aqueous batteries.

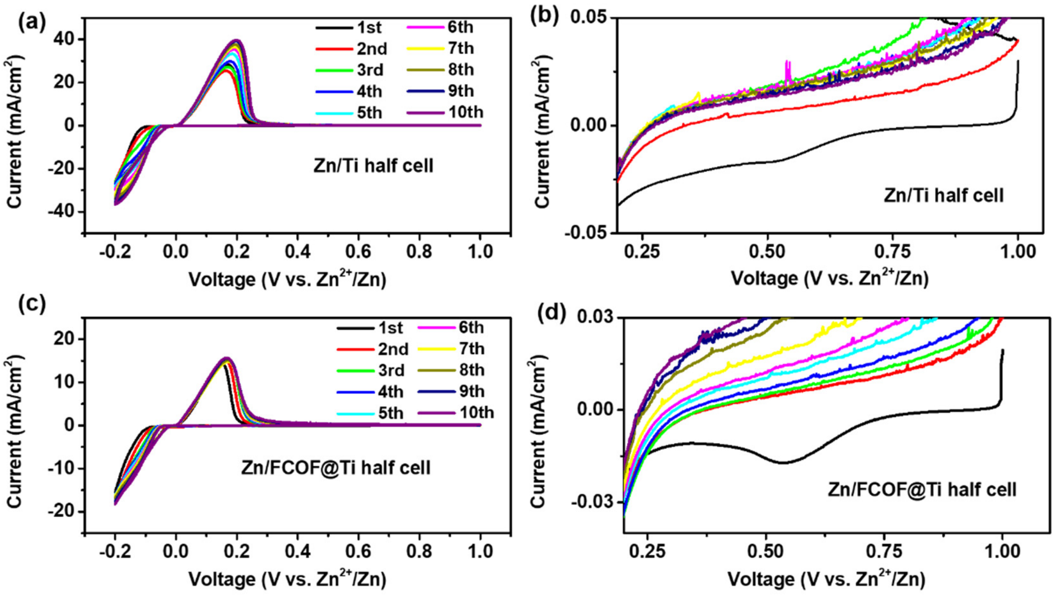

Supplementary Fig. 30: CV curves for FCOF@Ti/Zn and Ti/Zn half-cells at a scan rate of 1 mV s^{-1}. CV curves for **a.** Ti/Zn and **c.** FCOF@Ti/Zn cells over a scanning range of -0.2 to 1 V. Corresponding, enlarged CV curves for **b.** Ti/Zn and **d.** FCOF@Ti/Zn cells over the range 0.2 to 1 V.

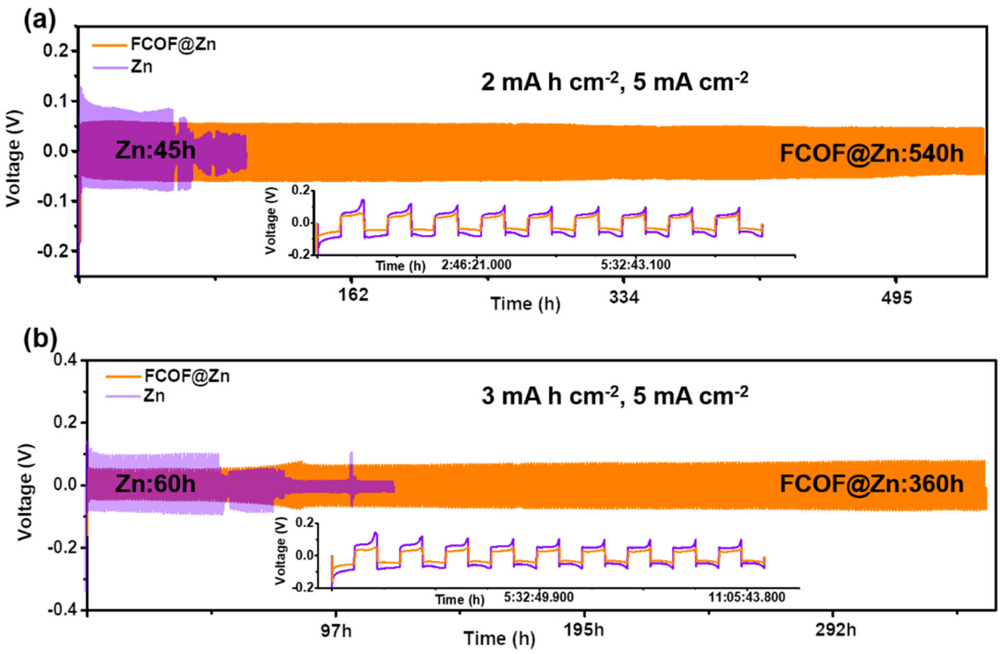

Supplementary Fig. 31: Cycling performance for Zn symmetric cells with/ without FCOF film protection. a. 2 mAh cm^{-2}, 5 mA cm^{-2}. **b.** 3 mAh cm^{-2}, 5 mA cm^{-2}. (Insets are initial voltage-time profiles).

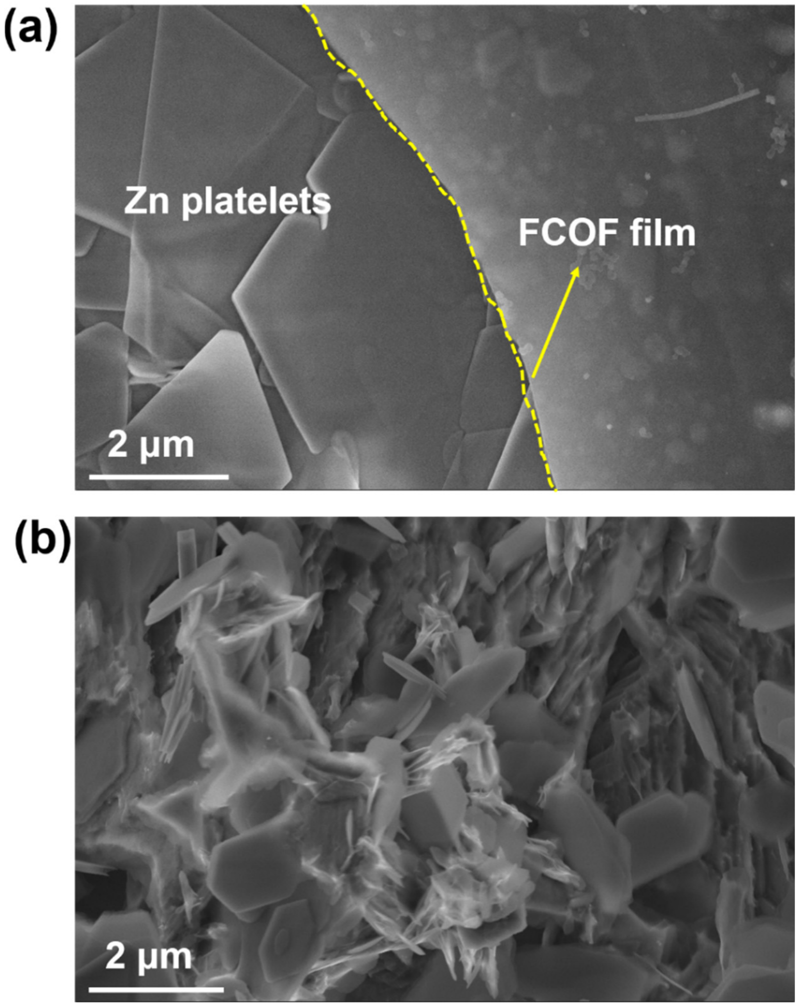

Supplementary Fig. 32: FESEM images of the Zn anode surface after long cycling.

The morphologies of **a.** FCOF@Zn and **b.** bare Zn anode.

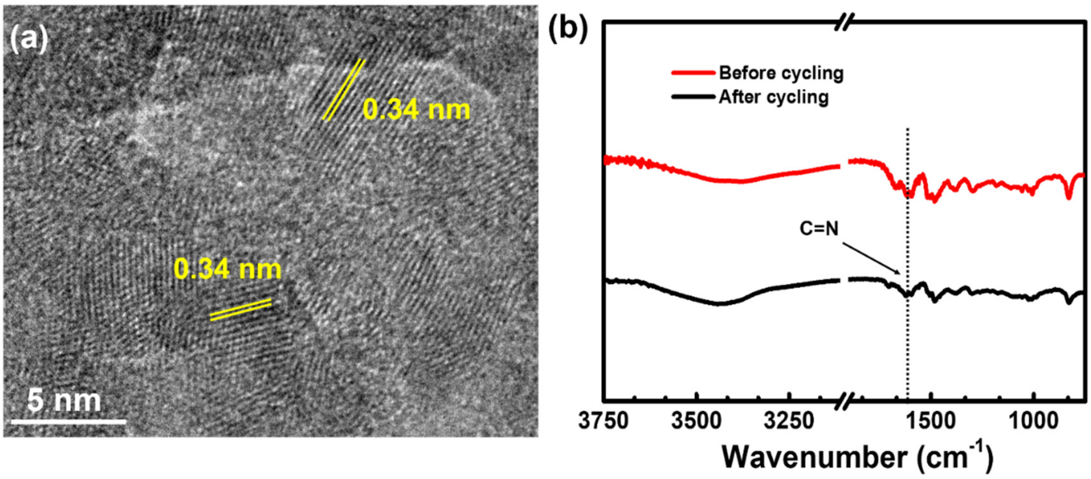

Supplementary Fig. 33: The characterization of the FCOF film after cycling. a. HRTEM image clearly shows the interlayer spacing of 0.34 nm, indicating the well-maintained 2D stacked structure. **b.** FTIR results before and after cycling show the obvious peak density of the C=N bond, demonstrating the strong stability of the covalent linked chemical structure in FCOF films.

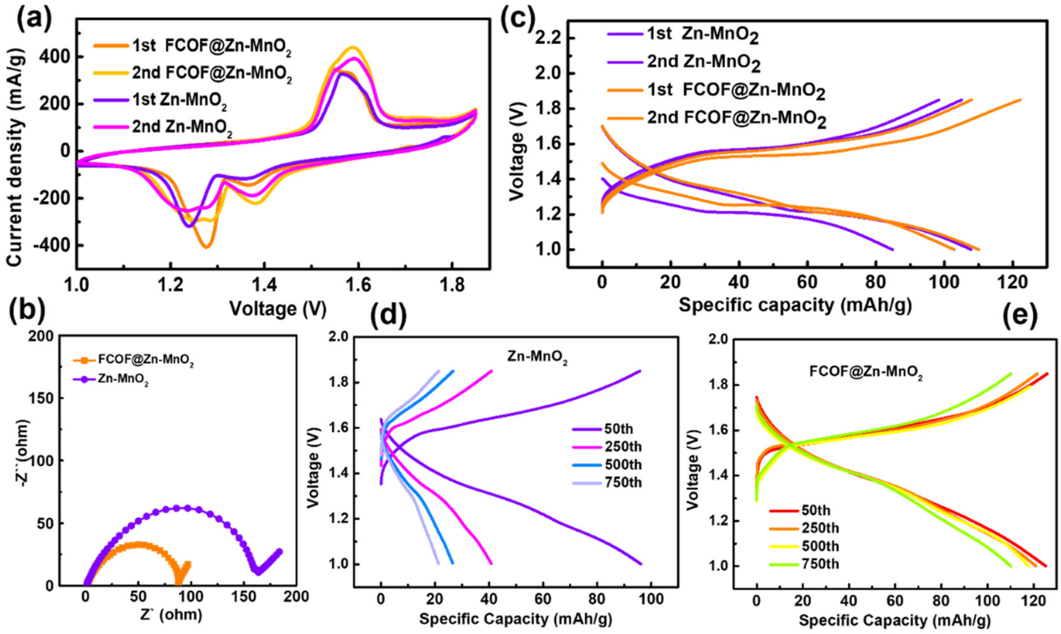

Supplementary Fig. 34: Electrochemical results of the full cells. a. 1st and 2nd CV curves of FCOF@Zn-MnO_2 and Zn-MnO_2 full cells. **b.** 1st and 2nd charge/discharge profiles of the FCOF@Zn-MnO_2 and Zn-MnO_2 full cells. **c.** EIS results of FCOF@Zn-MnO_2 and Zn-MnO_2 full cells. The 50th, 250th, 500th and 750th charge/discharge profiles of **e** FCOF@Zn-MnO_2 and **d.** Zn-MnO_2 full cells

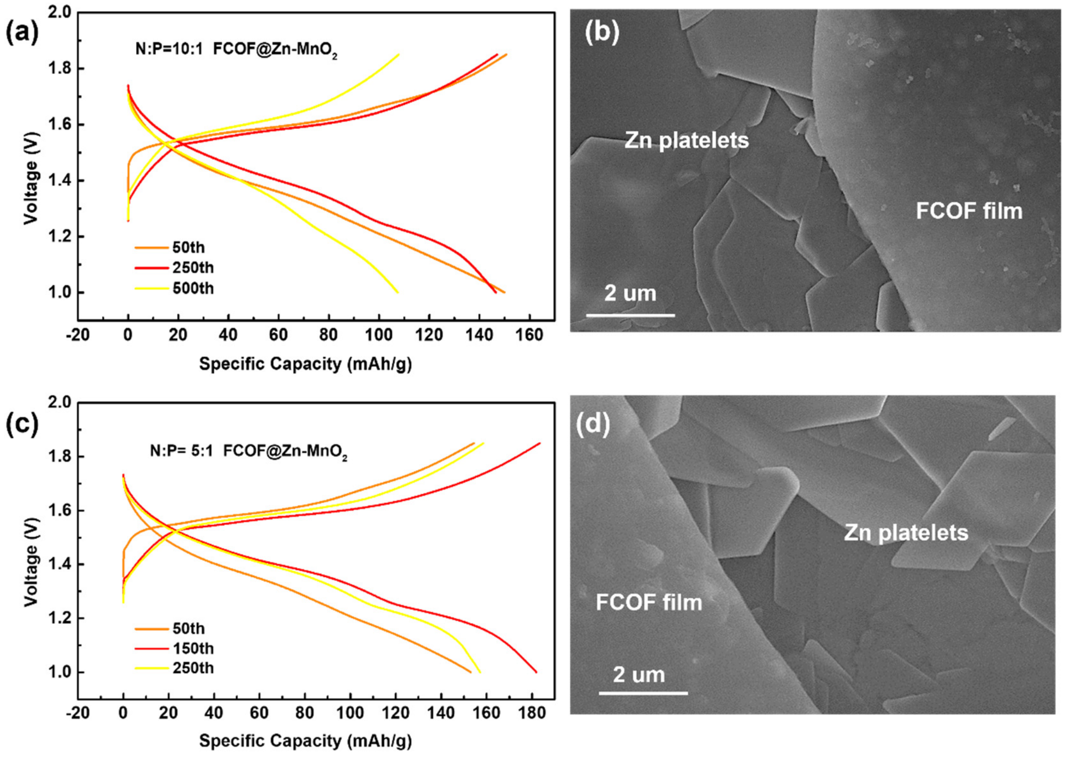

Supplementary Fig. 35: Electrochemical results of the FCOF@Zn-MnO_2 full cells at depth of discharge (DOD) of 10% and 20%, respectively. The charge/discharge profiles and FESEM images for FCOF@Zn anodes following long cycling at N/P ratio **a, b.** 10:1 and **c, d.** 5:1.

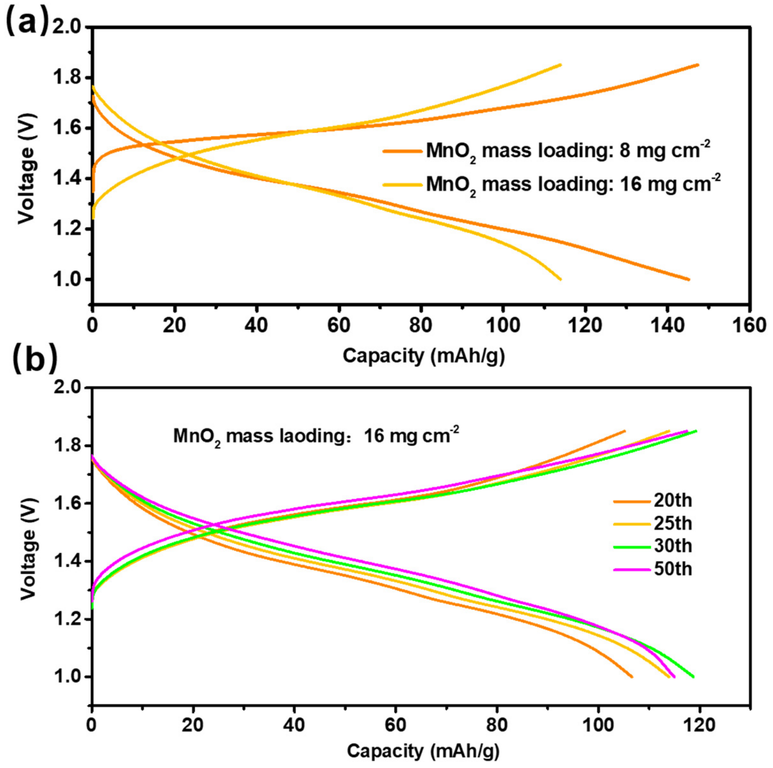

Supplementary Fig. 36: Charge/discharge profiles for FCOF@Zn-MnO_2 full cell

a. Different mass loadings collected from the 30th cycle and **b.** Low N:P capacity ratio of 2:1 with controlled electrolyte addition of 12 μL mA h^{-1}, current density 3 mA cm^{-2}.

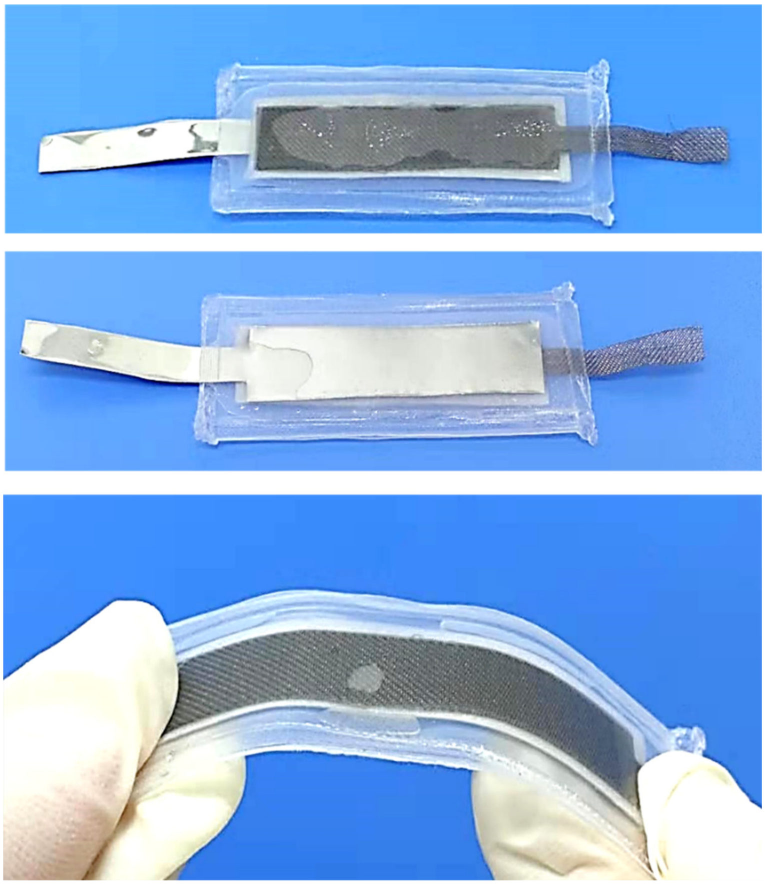

Supplementary Fig. 37: Pictures of the flexible transparent battery.

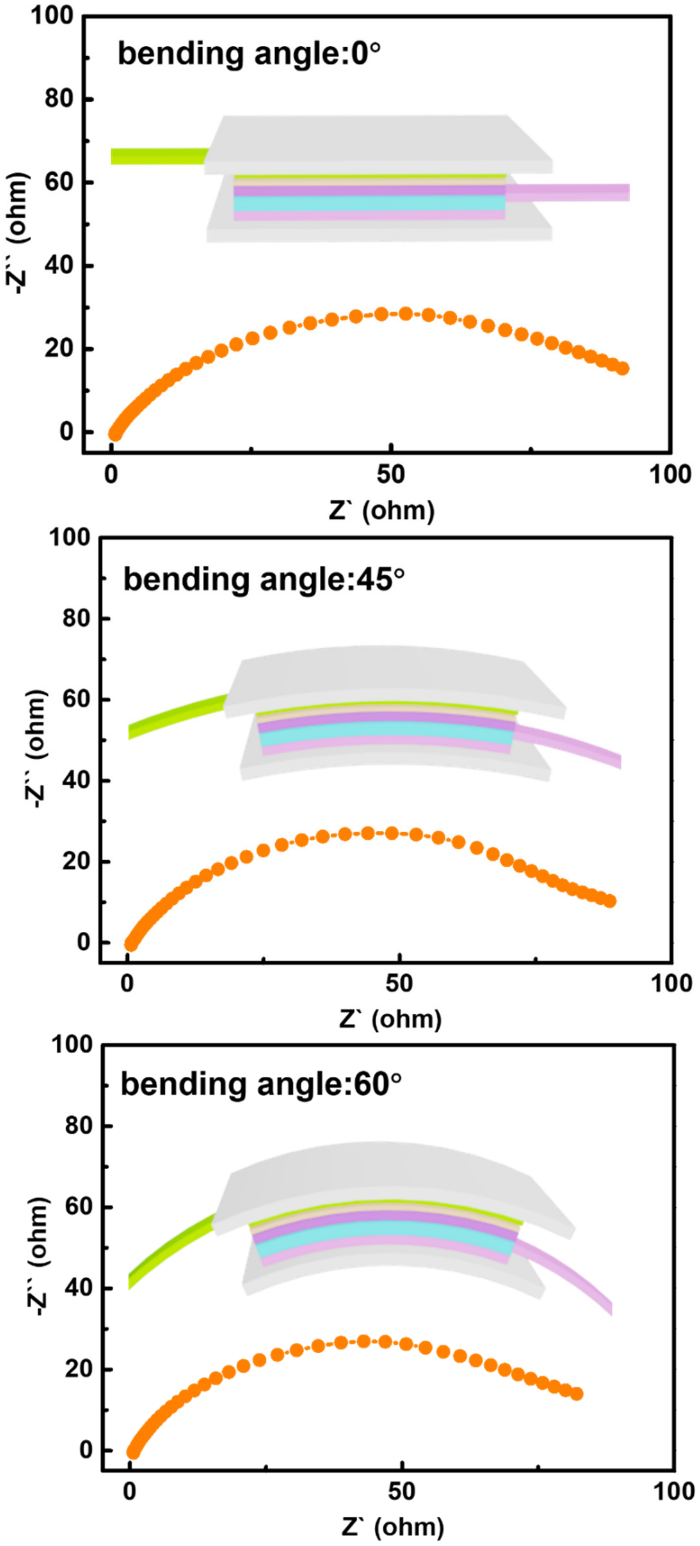

Supplementary Fig. 38: EIS results of the flexible battery at different bending angles.

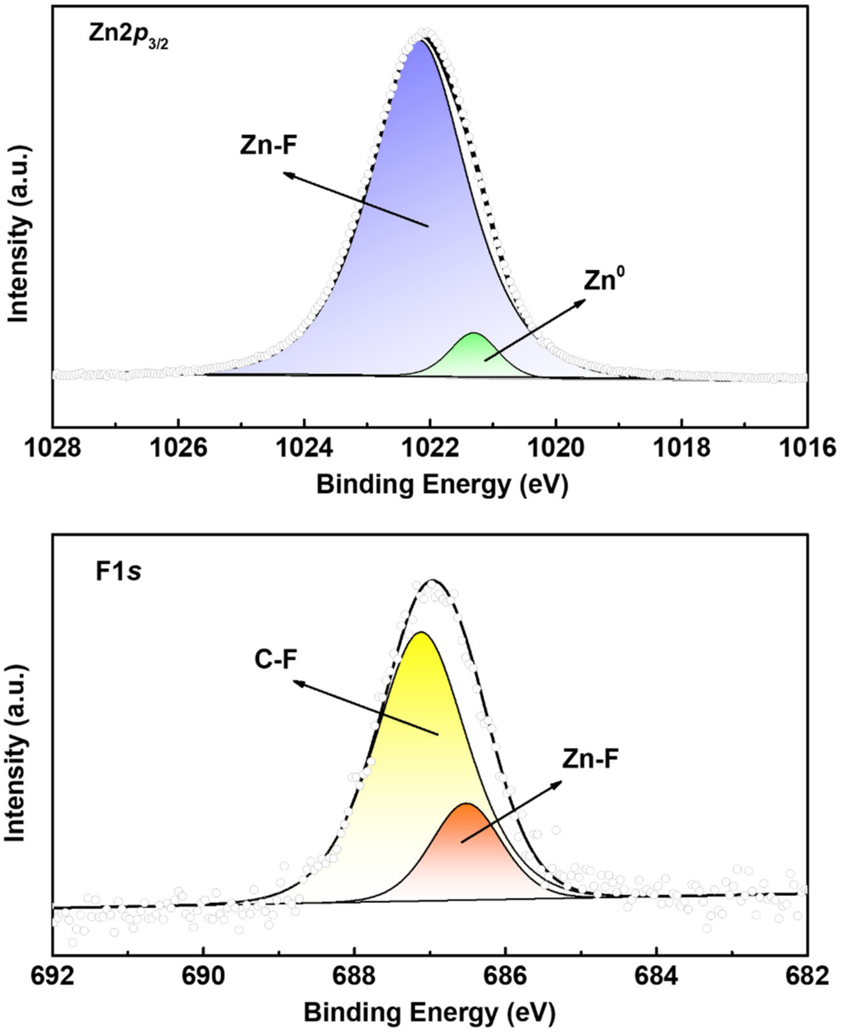

Supplementary Fig. 39: XPS results of the F 1*s* and Zn 2*p* at the FCOF-Zn interface.

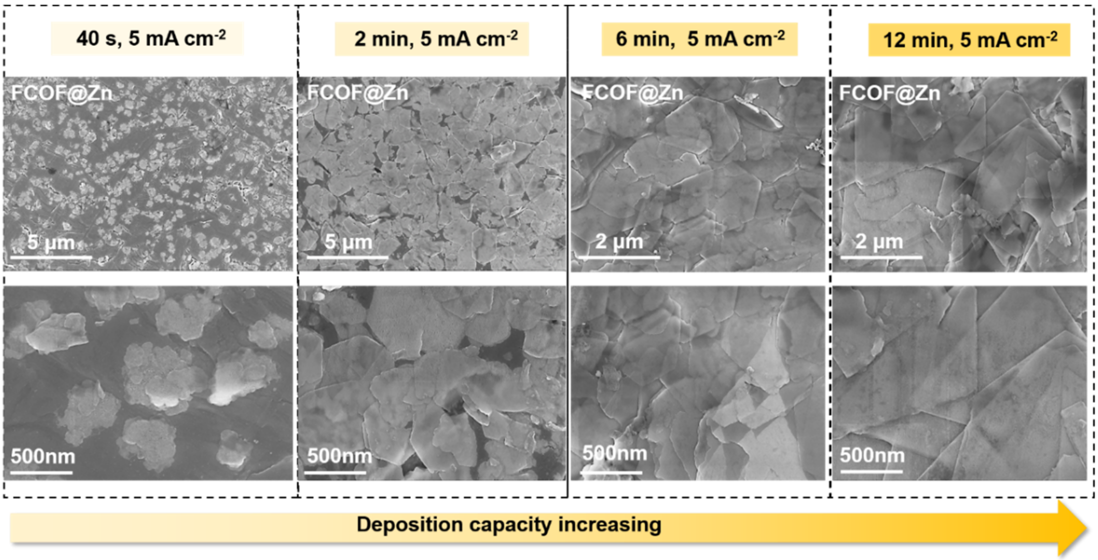

Supplementary Fig. 40: Evolution of the Zn deposition morphology underneath FCOF film.

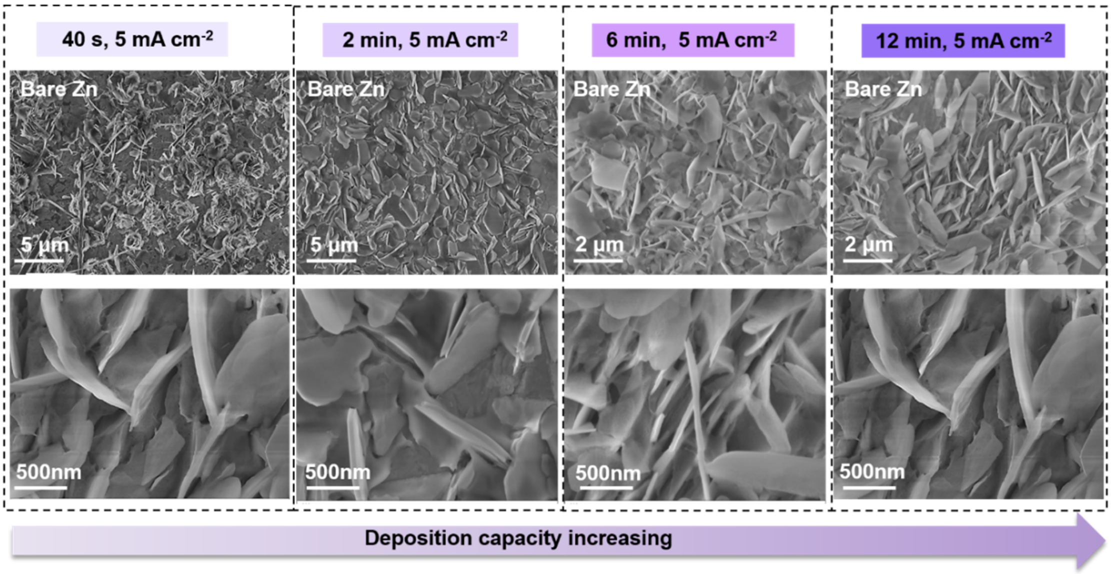

Supplementary Fig. 41: Evolution of the bare Zn deposition.

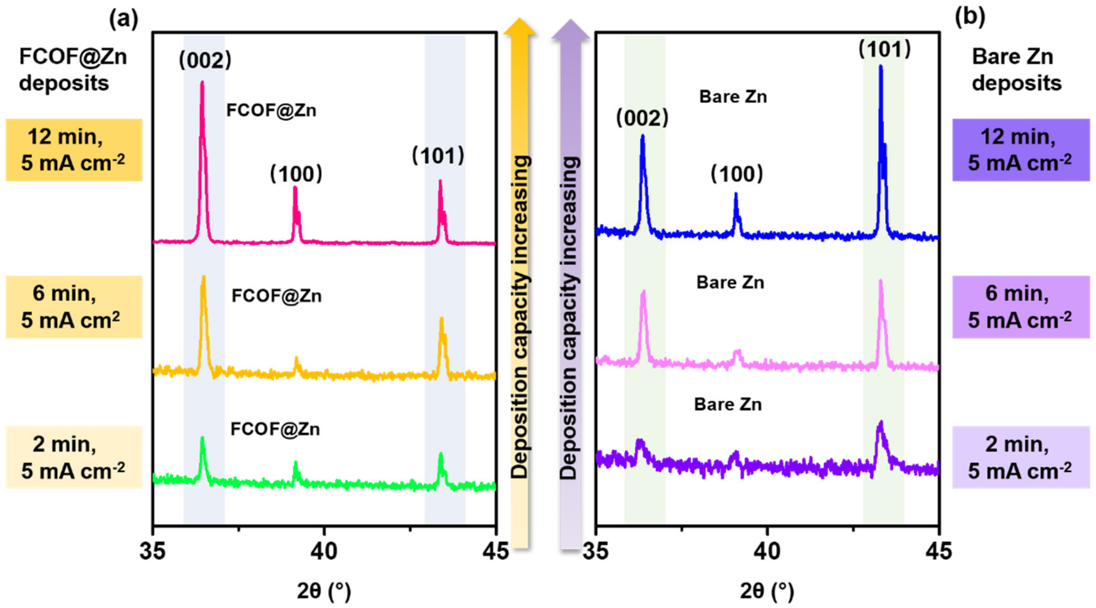

Supplementary Fig. 42: XRD patterns a. FCOF@Zn and **b.** Bare Zn, deposits.

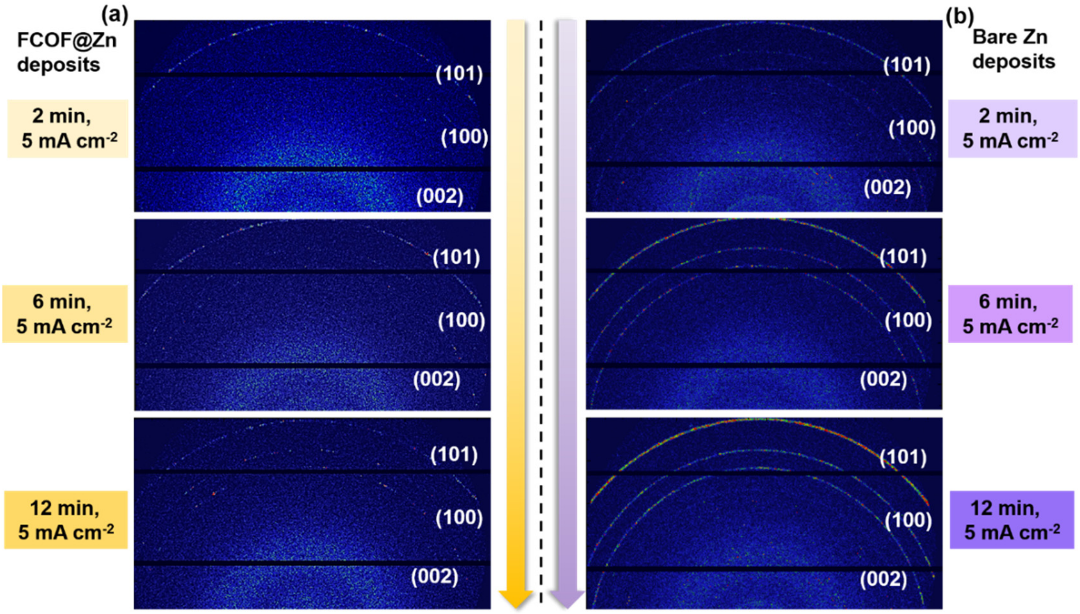

Supplementary Fig. 43: Two-dimensional WAXS patterns. a. FCOF@Zn deposits and **b.** Bare Zn deposits.
